# A unified multimodal model for generalizable zero-shot and supervised protein function prediction

**DOI:** 10.1093/bioinformatics/btag356

**Published:** 2026-06-04

**Authors:** Frimpong Boadu, Yanli Wang, Jianlin Cheng

**Affiliations:** Department of Electrical Engineering and Computer Science, University of Missouri, Columbia, Missouri 65211, United States; Roy Blunt NextGen Precision Health, University of Missouri, Columbia, Missouri 65211, United States; Department of Electrical Engineering and Computer Science, University of Missouri, Columbia, Missouri 65211, United States; Roy Blunt NextGen Precision Health, University of Missouri, Columbia, Missouri 65211, United States; Department of Electrical Engineering and Computer Science, University of Missouri, Columbia, Missouri 65211, United States; Roy Blunt NextGen Precision Health, University of Missouri, Columbia, Missouri 65211, United States

## Abstract

**Motivation:**

Predicting protein function is a fundamental and challenging task that requires integrating diverse biological data modalities to capture complex functional relationships. Traditional machine learning methods often rely on single modalities or combine only a limited number (typically two), without aligning them in a unified representation, thereby constraining predictive accuracy. Moreover, most existing machine learning approaches are limited to preselected subsets of Gene Ontology (GO) function terms with sufficient annotations, making the prediction of novel function terms a persistent challenge.

**Results:**

Here, we present FunBind, a multimodal AI model that jointly learns from five modalities, i.e., protein sequences, textual descriptions, domain annotations, structures, and GO terms, to enhance prediction accuracy and infer previously unseen functions. FunBind operates in two modes: (1) self-supervised pretraining using contrastive learning to align the sequence modality with other heterogeneous modalities in a unified latent space, enabling unsupervised zero-shot function prediction, and (2) supervised fine-tuning of the pretrained model to leverage all non-function modalities for comprehensive and accurate function classification. Our results show that FunBind’s zero-shot capabilities allow it to generalize effectively to novel function terms never encountered before, while its joint multimodal fine-tuning strategy outperforms single-modality models and current state-of-the-art deep learning methods in typical function prediction settings.

**Availability:**

https://github.com/jianlin-cheng/FunBind

## 1 Introduction

Accurate prediction of protein function is crucial for deepening our understanding of biological processes and advancing biomedical research and biotechnology development. Most traditional approaches (Yuan *et al*. 2023, Boadu and Cheng 2024, Boadu *et al*. 2025) rely on single-modality input data, such as protein sequences. However, single-modality methods (Boadu and Cheng 2024, Boadu *et al*. 2025) are unable to leverage functional information available across multiple complementary data modalities, limiting their ability to accurately predict the functions of many proteins.

There are substantial efforts to integrate a few modalities (mostly two) (Boadu *et al*. 2023, Kulmanov *et al*. 2024, Yuan *et al*. 2023, Yan *et al*. 2024), such as protein sequences and structures, to improve function prediction. However, these methods simply combine the features extracted from different modalities together without semantically aligning (or binding) them in a unified representation, leading to suboptimal performance. Moreover, these methods often require all the modalities to be available to make prediction, and therefore cannot be used when some modality is missing, severely limiting their applicability.

Recent advances have explored the use of contrastive learning and representation learning for multimodal protein function prediction. For instance, GALA (Fu *et al*. 2024) employs a generalized graph transformer to improve function prediction across dissimilar sequences, while GOBeacon (Lin *et al*. 2025) integrates contrastive learning with ensemble strategies to enhance protein function annotation.

Beyond the challenges of data integration, a significant obstacle in protein function prediction is the accurate identification of novel or poorly characterized function terms (for example, Gene Ontology (GO) terms (Aleksander *et al*. 2023)) that are absent or rarely represented in existing protein function datasets and databases. The prevalent use of supervised learning methods in the field, which depend on large, labeled datasets, limits the ability to predict novel function terms that have not been encountered during training and also results in poor performance on rare function terms.

To address this challenge, few-shot and zero-shot learning has emerged as promising solutions. Methods such as Tale (Cao and Shen 2021) and TransFew (Boadu and Cheng 2024) embed protein sequences and GO relationship information into a shared latent space, enabling the transfer of functional annotations from frequently annotated terms to rarely annotated ones. Similarly, DeepGOZero (Kulmanov and Hoehndorf 2022) and DeepGO-SE (Kulmanov *et al*. 2024) leverage logic axioms derived from the relationships between GO terms to enhance rare or novel protein function prediction. Additionally, ProTranslator (Xu and Wang 2022) integrates protein sequence data, protein-protein interactions, and textual descriptions to enhance predictions for rare function terms. ProtNote (Char *et al*. 2025) leverages pretrained language models and textual descriptions of GO terms to enable zero-shot protein function prediction via embedding-based similarity.

While these approaches improve the prediction of rare GO terms, they still have limitations. Many of them rely on fixed vector representations of protein function terms, which restrict their ability to generalize beyond predefined functional terms in the training data. Therefore, they still cannot accurately predict novel GO terms never seen during training. Moreover, their dependence on supervised training introduces biases linked to the quality and quantity of available labeled data, ultimately constraining their applicability to poorly annotated function terms.

To address these challenges, we introduce FunBind, a novel multimodal AI model to integrate protein sequences, structures, textual descriptions, and domain annotations for robust protein function prediction. Unlike previous approaches, FunBind effectively operates in scenarios with missing modalities by leveraging protein sequences as the central modality for cross-modality alignment, ensuring a stable foundation for learning. Given their near-universal availability, sequences provide a reliable anchor for integrating any additional data sources, even when certain modalities are missing.

Furthermore, FunBind incorporates self-supervised pretraining via contrastive learning to enable unsupervised zero-shot function prediction, allowing it to generalize to unseen GO terms without explicit training examples. This capability is crucial for annotating novel or poorly characterized proteins, where traditional supervised methods often fail. By learning a unified joint embedding space, FunBind captures the inherent relationships between modalities, enabling effective extrapolation to previously unseen functional terms.

We conduct extensive experiments, including contrastive learning-based multimodal alignment (binding), self-supervised pretraining, zero-shot function prediction, and supervised fine-tuning for function classification to rigorously assess FunBind’s performance. Our results demonstrate that FunBind outperforms current state-of-the-art approaches in protein function classification by effectively leveraging diverse, complementary data modalities, even when some modalities are absent. Moreover, FunBind can predict novel function terms that were never seen during training, highlighting its ability to make biologically meaningful zero-shot predictions.

## 2 Methods

### 2.1 Datasets

We collected protein sequences (*sequence modality*) and their functional annotations (*GO term labels modality*) from the fifth Critical Assessment of Functional Annotation (CAFA5) (Zhou *et al*. 2019) dataset, which primarily includes annotations released by November 2022, for model training and validation. Only sequences associated with at least one additional non-function modality (structure, text, or Interpro domain annotation) were retained.

The predicted structures (*structure modality*) for the proteins were retrieved from AlphaFoldDB (Jumper *et al*. 2021), specifically the swissprot_pdb_v4.tar subset, and converted into one-dimensional (1D) structural representations (known as 3Di strings) using FoldSeek (Van Kempen *et al*. 2024). These 3Di strings were used to generate structural embeddings.

Text descriptions (*text modality*) of the proteins were sourced from UniProt/Swiss-Prot (UniProt Consortium 2019), focusing on curated fields such as subcellular location, subunits, functions, induction, tissue specificity, and similarity.

InterPro domain annotations (*Interpro modality*) were extracted from the InterPro database (Paysan-Lafosse *et al*. 2023) for the proteins with available information. The statistics for the training data is presented in Table S1, available as supplementary data at *Bioinformatics* online, which details the number of proteins in three functional categories: CC (Cellular Component), MF (Molecular Function), and BP (Biological Process) and all function categories combined for each non-function modality.

To construct the test datasets, we collected newly annotated proteins from UniProt (specifically the goa_uniprot_all.gaf set), released on December 21, 2024. The GO terms were obtained from UniProt, while the Gene Ontology graph was sourced from the Gene Ontology Resource (Aleksander *et al*. 2023). To compile the complete set of GO terms (labels) for each protein, we first retrieved its direct annotations from UniProt and then expanded this set by traversing the GO graph to include all ancestor terms.

Following the guidelines established in CAFA (Zhou *et al*. 2019), we retained only annotations supported by strong evidence codes (EXP, IDA, IPI, IMP, IGI, IEP, TAS, IC, HTP, HDA, HMP, HGI, HEP), ensuring that each protein is labeled with high-confidence functional information. We refer to this curated dataset of newly annotated proteins as **Test_All** (see its statistics in Table S2, available as supplementary data at *Bioinformatics* online. The text modality and Interpro annotation modality of the proteins in Test_All are based on the text descriptions released by December 2022, prior to the release of their function labels in 2024, to prevent any functional information leak.

Moreover, the proteins in Test_All that have less than 30% sequence identity with any proteins in the training data were used to create a dataset—**Test_Novel**, (see its statistics in Table S3, available as supplementary data at *Bioinformatics* online). Test_All and Test_Novel were used to compare the fined-tuned FunBind and existing methods.

To evaluate zero-shot function prediction, we constructed another test set **(Test_Zero)** from Test_All. We collected all proteins that have at least one novel GO term that does not appear in the training data. This ensures that the evaluation measures the model’s ability to generalize to novel GO terms never encountered during training. The Test_Zero data includes 659 proteins. Among them, 450 proteins have one new annotation (GO term), 156 have two, 21 have three, 24 have four, and 8 have five to six new annotations. Notably, protein A8BPK8 has 17 new annotations. The statistics of Test_Zero is shown in Table S4, available as supplementary data at *Bioinformatics* online.

### 2.2 Architecture of FunBind

**Figure 1:**
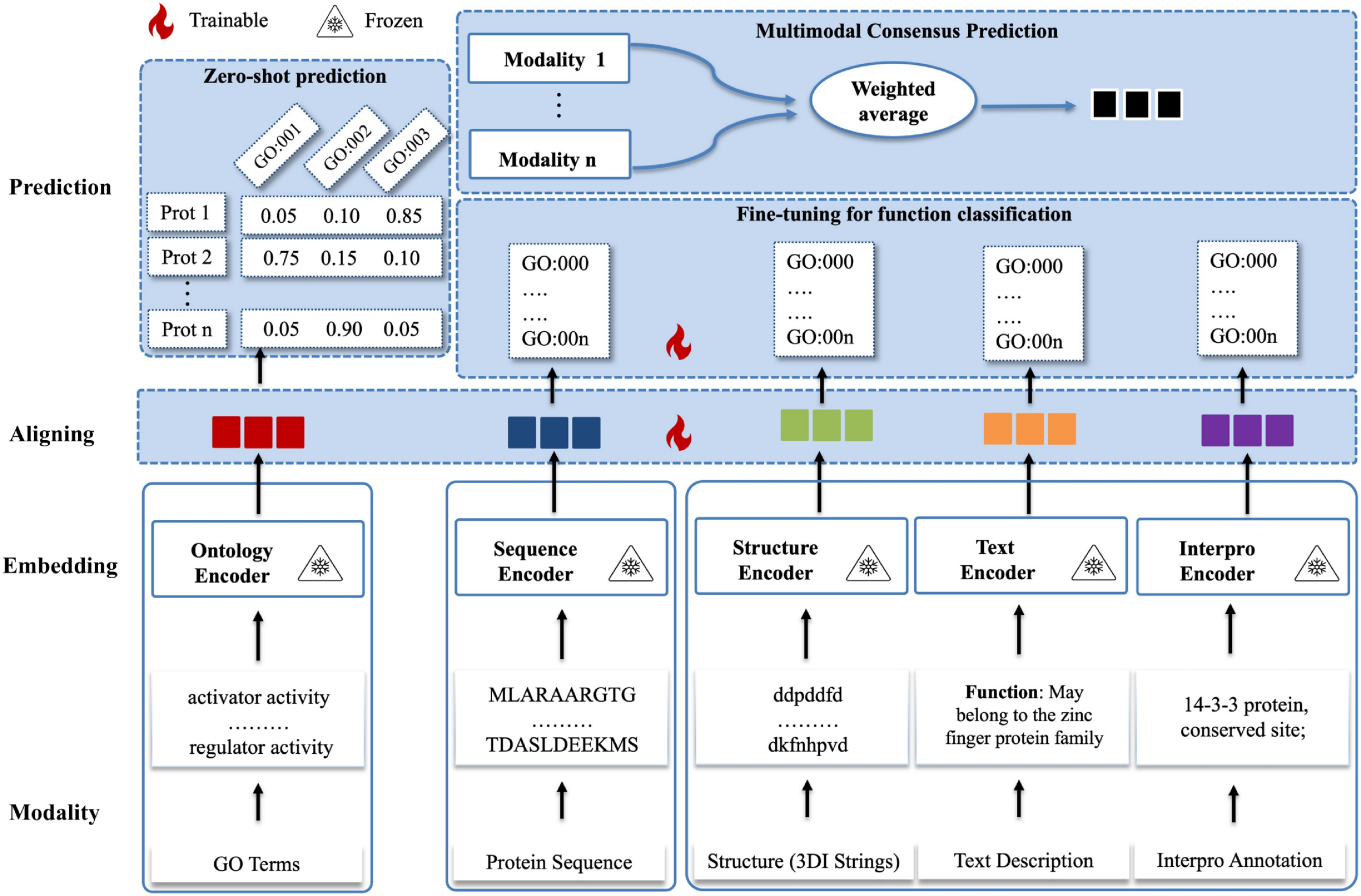
The architecture of FunBind. It integrates five data modalities including protein sequences, structures represented as 3Di strings of FoldSeek, text description, InterPro domain annotations, and GO function terms via self-supervised contrastive learning. Each modality is first processed through a specialized frozen encoder to generate an embedding. The embeddings are aligned in the latent space during pretraining. After the pretraining, it can be directly used to make zero-shot prediction of protein function by evaluating the embedding of each GO term candidate against the sequence modality (or other non-function modality) of a protein. Moreover, the aligned embedding of each non-function modality of proteins generated by the pretrained model can be used by a multi-label classification head to predict their function through joint supervised fine-tuning. The predictions from the non-function modalities are then fused by weighted averaging to generate the multimodal consensus function prediction.

Inspired by the vision-language model (e.g. CLIP) of aligning images and texts (Radford *et al*. 2021, Girdhar *et al*. 2023), we develop a multi-modal AI model (FunBind) to align five modalities of proteins—including sequences, structures, text description in UniProt, domain annotations in the InterPro database (Paysan-Lafosse *et al*. 2023), and GO function terms—in the latent space by contrastive learning, i.e., maximizing the similarity between the embeddings of modalities of the same proteins and minimizing the embedding similarity of different proteins (Fig. 1).

After the model is pre-trained by self-supervised contrastive learning, it can be directly used to predict the probability of any function term for a protein by evaluating how closely the embedding of the term is aligned with any other modalities of the protein (e.g., sequence), enabling the unsupervised zero-shot prediction of novel function terms. Moreover, FunBind is further fine-tuned via multi-label supervised learning to integrate the embeddings of all the available non-function modalities to predict function for proteins. Both the self-supervised contrastive learning and the multi-label supervised fine-tuning can work with any number of modalities, even when some modalities are missing.

The objective of pretraining is to learn a unified embedding space for the five modalities, with the protein sequence modality serving as the central modality for cross-modality alignment. This is because the protein sequence is always available, while other modalities may be missing. By aligning (binding) all other four modalities with the sequence modality, the model can leverage the sequence information to learn relationships between different modalities. This approach contrasts with traditional methods that simply combine modalities without leveraging such relationships. Additionally, this strategy simplifies the learning process by requiring only n−1 modality alignments rather than n×(n−1) pairwise alignments (*n*: number of modalities and *n* = 5 here).

FunBind’s architecture (Fig. 1), comprises three core modules: (a) Modality encoding module to process input data from different modalities to generate embeddings, (b) Modality alignment (binding) module, to align embeddings across modalities in the latent space, and (c) Prediction module, to output functional annotations based on learned relationships, which has two sub-modules: (1) unsupervised zero-shot prediction and (2) supervised multi-label classification-based prediction.

For the zero-shot function prediction, the pretrained model predicts GO terms solely from alignments between a non-function modality and a GO term modality (e.g., sequence-GO alignments), i.e., the GO terms that align best with the sequence modality or other modalities are selected as predicted function terms. This unsupervised approach can predict any GO function terms including novel ones never encountered before.

For the multi-label classification-based function prediction, the pretrained model is further fine-tuned by supervised learning to use available non-function modalities to predict function. Here, the aligned embedding of each available non-function modality is used by a multi-label classification head to predict protein functions within a fixed set of pre-determined GO terms that serve as classes. The predictions from all available non-function modalities are averaged to generate the multimodal consensus prediction. The details of each module of FunBind are described in the subsections below.

### 2.3 Modality encoding

FunBind begins with an input modality encoding block, which generates initial embeddings for each protein by applying a modality-specific encoder to every available modality (Fig. 1).

For protein sequence modality, we tested two pretrained protein language models: ESM2-t48 (Lin *et al*. 2022) and ProstT5 (Heinzinger *et al*. 2024). While ProstT5 demonstrated better performance in binding sequence embeddings to structural embeddings, ESM2-t48 achieved stronger results when aligning sequence embeddings with text and InterPro modalities, as shown in Fig. S1, available as supplementary data at *Bioinformatics* online. We trained separate models for each encoder and compare their performance (Table S5, available as supplementary data at *Bioinformatics* online). Since the overall differences is minor, ESM2-t48 is used as the default sequence encoder for the final model.

For protein structure modality, we employ FoldSeek (Van Kempen *et al*. 2024) to convert three-dimensional structures into 1D structural representations known as 3Di strings consisting of 3Di-tokens. These structural sequences are then encoded using the ProstT5 structural module, producing embeddings enriched with structural context.

Inspired by ProtST (Xu *et al*. 2023), we construct text modality by aggregating information from diverse protein-related fields in UniProt, including protein names, functional descriptions, subcellular locations, subunits, induction, tissue specificity, and similarity. These fields are concatenated into a single textual sequence for each protein. The statistics of the individual fields and the results of an ablation study assessing their contribution to GO term prediction performance are provided in Supplementary Tables S6 and S7, available as supplementary data at *Bioinformatics* online respectively. The ablation study shows that the Function field has the largest overall impact on prediction performance, while Subcellular Location and Similarity are particularly important for CC and MF, respectively. In contrast, Tissue Specificity and Induction contribute the least, as their removal has minimal effect.

Similarly, each protein’s InterPro annotation modality in text format is generated by combining the name, description, and the set of mapped InterPro domains, as illustrated in Fig. S2, available as supplementary data at *Bioinformatics* online.

For GO term modality, we collect the textual definitions of GO terms of a protein spanning the three function categories: Cellular Component (CC), Molecular Function (MF), and Biological Process (BP). For each protein, we sample a subset of GO terms—preferably leaf nodes—in the GO graph, and construct textual representations by concatenating their namespaces, GO term names, and definitions, as depicted in Fig. S2, available as supplementary data at *Bioinformatics* online.

To encode both the InterPro modality and the text modality, we compared LLaMA2 (Touvron *et al*. 2023), a large autoregressive language model, with BioBERT (Lee *et al*. 2020), a transformer model pre-trained specifically on biomedical literature. Based on its superior performance across modalities shown in Fig. S1, available as supplementary data at *Bioinformatics* online, we select LLaMA2 as the encoder for the two modalities. Similarly, for generating the embedding of the GO term modality, we use LLaMA2 to encode the textual descriptions of GO terms.

When using LLaMA2, each input annotation is tokenized with an End-of-Sequence (EOS) token appended to mark the end, and sequences are padded to ensure consistent batching. We set the max_length parameter to 1024 tokens for all three modalities (Interpro, text, and GO term), as most input sequences fall within this range. The distribution of sequence lengths is shown in Fig. S3, available as supplementary data at *Bioinformatics* online.

To reduce computational costs, we kept the weights of all base encoders (ESM, ProstT5, and LLaMA2) frozen throughout the training of FunBind. This design choice allows it to focus on learning effective cross-modality alignments and function prediction.

### 2.4 Modality alignment (binding)

The modality alignment and integration module aims to learn a unified embedding space that facilitates cross-modality alignment. We employ a contrastive learning approach with pairs of modalities (S, M) to learn modality alignment, where S represents the protein sequence modality and M represents any other modality. In our experiments, we bind structure, text, InterPro domain annotation, and GO term modalities to the central sequence modality.

The objective is to maximize the similarity between paired modalities from the same proteins in the embedding space while minimizing the similarity between unpaired modalities from different proteins, thereby encouraging modality embeddings to align when semantically related.

Given a protein *i*, with sequence $S_{i}$ and another modality $M_{i}$, we generate embeddings as follows: $q_{i}=f(S_{i})$ and $k_{i}=g(M_{i})$, where *f* and *g* are modality-specific projection networks applied to the representations obtained during the encoding phase using the pre-trained encoders (i.e., language models). These projection layers transform the modality-specific embeddings into a shared embedding space suitable for contrastive learning.

To capture modality-specific nuances and enable effective cross-modality integration, we employ a Mixture of Experts (MoE) architecture for the projection of each input modality. Each MoE comprises 1 to 3 parallel experts, with each expert implemented as a stack of multilayer perceptrons (MLPs) interleaved with Batch Normalization, GELU (Gaussian Error Linear Unit) activations, and Dropout layers.

The outputs of all experts are combined using a gating mechanism, which dynamically computes a weighted combination of the expert outputs based on the input. This gating network assigns higher weights to experts that are more relevant for a given input, enabling the model to flexibly adapt to different data patterns within each modality. As a result, each expert can specialize in capturing distinct features, while the gating mechanism ensures that the final representation is tailored to the specific characteristics of the input (as illustrated in Fig. S4, available as supplementary data at *Bioinformatics* online).

To promote the learning of complementary representations and prevent experts from collapsing to similar functions, we introduce a diversity loss. This loss encourages the gating network to activate experts in a way that maximizes variation among their outputs. It is defined as:


(1) $$L_{\text{diversity}}=1+\frac{1}{N(N-1)}\sum_{i=1}^{N}\sum_{j=i+1}^{N}\;\text{cos}\;(E_{i},E_{j})$$

Where $E_{i}$ and $E_{j}$ are the output embeddings of experts *i* and *j*, and $\text{cos}(E_{i},E_{j})$ denotes their cosine similarity.

This loss penalizes similarity between expert outputs—growing larger when embeddings are highly similar and smaller when they are dissimilar or negatively correlated. It encourages each expert to specialize in different aspects of the input, thereby enriching the model’s ability to capture diverse, modality-specific representations.

### 2.5 Self-supervised pretraining

During self-supervised pretraining, we optimize the embeddings generated by the MoEs using an InfoNCE loss for contrastive learning (Radford *et al*. 2021), regularized by the diversity loss term that ensures the MoEs produce diverse representations. The InfoNCE loss($L_{S,M}$) is defined as:


(2) $$L_{S,M}=-\text{log}\;\frac{\;\text{exp}\;\left(s_{i}^{⊤}m_{i}/\tau \right)}{\;\text{exp}\;\left(s_{i}^{⊤}m_{i}/\tau \right)+\sum_{j\neq i}\;\text{exp}\;\left(s_{i}^{⊤}m_{j}/\tau \right)}$$

Where τ is a scalar temperature controlling the smoothness of the softmax, $s_{i}$ represents the embedding of protein *i* from the sequence modality. $m_{i}$ represents the embedding of the same protein *i* from non-sequence modality *M*, $m_{j}$ represents the embedding of a different protein *j* (where j≠i) from modality *M* serving as a negative sample.

The final loss function, $L_{\mathrm{total}}$, is a combination of the InfoNCE loss and the diversity loss:


(3) $$\mathrm{Loss}=L_{S,M}+\lambda L_{\text{diversity}}$$

Where λ is a hyperparameter that controls the weight of the diversity loss term.

### 2.6 Unsupervised zero-shot function prediction

After FunBind is pretrained, it can be directly used to make zero-shot prediction of protein function terms from any non-function modality (e.g., sequence and text) by calculating the cosine similarity between the embeddings generated for candidate GO terms and the embeddings of the other modality of proteins. The similarity can be used to rank GO terms for a protein. Top *k* GO terms can be used as predictions, no matter if they were encountered during the training or not. Therefore, FunBind can predict novel GO terms never seen before.

### 2.7 Supervised function classification via fine-tuning

The pretrained FundBind can also be fine-tuned to predict protein function (GO terms) from one or multiple available non-function modalities. Building on the pretrained multimodal backbone, we extend the model to perform supervised multi-label function classification for the three GO function categories: CC, MF, and BP. Different from the open-ended unsupervised zero-shot prediction that works for any GO terms including novel ones, the supervised classification requires the number of function classes (GO terms) is predetermined. Like existing supervised function prediction methods, to mitigate label sparsity and focus on informative GO terms, we limit the prediction space to the frequently annotated GO terms in the training data. Specifically, we retain GO terms with at least 20 annotations for CC and MF and a higher threshold of 250 annotations for BP, resulting in 1043 classes for CC, 1529 classes for MF, and 1631 classes for BP.

To support this supervised learning task, we augment the pretrained foundational model backbone with a dedicated classification head for each non-function modality. Each classification head is composed of a series of multilayer perceptrons (MLPs) interleaved with Batch Normalization, GELU activation functions, and Dropout layers. The final layer outputs logits for the GO terms for each specific ontology (CC, MF or BP) and applies a sigmoid activation to enable multi-label prediction of GO terms. Because proteins can have a varied number of input modalities, the model generates one separate prediction for each available non-function modality.

These classification heads allow the general-purpose features learned during the pretraining to be specialized for the gene ontology-specific function prediction.

We jointly optimize (fine-tune) all classification heads using the binary cross-entropy (BCE) loss, formulated as:


(4) $$L_{\text{total}}=\sum_{M\in M}\left(\text{BCE}(S)+\text{BCE}(M)\right)$$

Where M={structure,text,InterPro} denotes the set of non-sequence modalities. In each training epoch, we iterate over each sequence–modality pair (S,M) and accumulate their losses. BCE(S) is recomputed independently for each sequence–modality pair.

At inference time, the model supports flexible prediction using any single modality or a combination of available modalities. This design allows it to handle varying levels of data completeness and leverage multiple sources of information when available for more robust predictions. While there are many possible strategies for combining the predictions of individual modalities, in this work, we adopt a simple weighted averaging scheme to combine individual predictions. Specifically, we assign equal weights of 0.25 to each of the four modalities and combine their predictions through weighted averaging.

## 3 Results

### 3.1 Zero-shot prediction of protein function using sequence modality

For zero-shot function prediction, the pretrained FunBind first generates aligned embeddings for protein sequences and any GO terms under consideration in a shared latent embedding space, then computes cosine similarity between the embeddings, and finally assigns the GO terms with the highest similarity scores as predicted functions for the proteins. The zero-shot function prediction was tested on the Text_Zero dataset.

The test proteins in the Test_zero dataset were randomly divided into groups (batches), each containing 10–12 proteins and the same number of novel GO terms never encountered during training. Each protein was associated with a single novel GO term, which was used as its label. Within each group, all the protein sequences were aligned with all candidate GO terms to calculate the similarity between each protein sequence and each GO term, which was used to rank GO terms for each protein. This setup is similar to how the zero-shot prediction was evaluated with vision-language models (Radford *et al*. 2021).

To account for variability in random grouping, the experiment was repeated 10 times. In each trial, proteins were randomly grouped, ensuring that the results reflect the model’s performance across different groupings and reducing the potential bias from random selection. The mean and standard deviation of performance metrics are reported for each function category as well as for the All function categories in Table 1. The performance is evaluated using Recall@k (R@1, R@3, R@5), which measures the fraction of proteins with correctly retrieved novel GO terms among the top-k predictions, and Mean Reciprocal Rank (MRR), which considers the ranking position of relevant GO terms.

**Table 1:** The performance of zero-shot function prediction using sequence modality as query.

Ontology	R@1	R@3	R@5	MRR
CC	0.7548	0.8732	0.9129	0.8302
	(±0.0254)	(±0.0124)	(±0.0201)	(±0.0129)
MF	0.6357	0.8405	0.9048	0.7550
	(±0.0564)	(±0.0302)	(±0.0213)	(±0.0312)
BP	0.4821	0.6869	0.7845	0.6225
	(±0.0374)	(±0.0160)	(±0.0180)	(±0.0206)
All	0.5947	0.7969	0.8749	0.7180
	(±0.0280)	(±0.0111)	(±0.0105)	(±0.0161)

The mean and standard deviation of the recall at 1, 3, and 5 predictions, and Mean Reciprocal Rank (MRR) for 10 trials on the proteins in Test_Zero dataset.

As shown in Table 1, across all three function categories (CC, MF, and BP), recall improves as more predictions per protein are considered. The recall for one prediction per protein (R@1) ranges from 0.4821 for BP, 0.6357 for MF, to 0.7548 for CC, and increases to 0.7845 for BP, 0.9048 for MF, and 0.9129 for CC, when making five predictions per protein (R@5). These results demonstrate FunBind can rather accurately predict novel GO terms not encountered during training.

Among the three GO function categories, CC exhibits the highest retrieval performance across all metrics, followed by MF and then BP. This performance disparity may be partly due to the level of the clarity in the definition of the GO terms in each ontology. CC and MF terms at the same level in the gene ontology graph may be more distinct from each other than BP terms, making them easier to differentiate. For example, two different CC terms at the same level refer to two completely different cellular locations without any overlap, while some BP terms may have some semantic similarity.

To illustrate how the zero-shot function prediction selects GO terms for a protein, Fig. S5, available as supplementary data at *Bioinformatics* online visualizes the protein sequence–GO term alignment similarity. Each heatmap visualizes the similarity between the proteins and the novel GO terms in a group. The proteins and their corresponding GO terms are arranged sequentially in the same order, with each element on the diagonal of the heatmap representing the similarity score between each protein and its corresponding true GO term (label) and each element off the diagonal representing the similarity score between each protein and a GO term not associated with it. It is shown that many elements on the diagonal have relatively higher similarity scores, highlighting the strong alignment between protein sequences and their labels, which leads to a correct prediction. It is worth noting that the zero-shot prediction of novel function terms is done fully through the unsupervised contrastive learning without using label-based supervised learning employed by most machine learning methods for function prediction.

### 3.2 Zero-shot function prediction using other non-central modalities

During the pretraining of FunBind, protein sequence serves as the central modality to be aligned with other modalities of proteins (text, structure, InterPro, and GO terms). There is no direct alignment between GO terms and other non-sequence modalities. However, we hypothesize that the other non-sequence modalities and GO terms have been aligned implicitly through the bridge of the central sequence modality, a phenomenon known as emergent binding in the vision-language models (Girdhar *et al*. 2023).

To investigate this emergent binding, we evaluated FunBind’s zero-shot prediction of using structure modality, text modality, and InterPro modality as queries to predict GO terms, respectively. This evaluation follows the same methodology as the sequence-based zero-shot prediction, where the cosine similarity between modality embeddings and GO term embeddings is used to rank and select GO terms for a protein. The performance of zero-shot function prediction using each non-sequence modality was tested on the same test proteins used in the sequence-based zero-shot evaluation, which have all five data modalities available.

The structure modality consistently achieves competitive scores across all metrics and ontologies in zero-shot prediction Table S8, available as supplementary data at *Bioinformatics* online. It slightly outperforms the text modality in MF and BP considering all the metrics while remaining close to it in CC. Both of them generally perform better than Interpro modality in all three function categories. The text modality is the strongest in CC, reaffirming its utility when available. The InterPro modality performs comparably to the text modality in MF but lags slightly in CC and BP, indicating that domain annotations are informative yet less comprehensive than the full textual descriptions. Compared to the central sequence modality (Table 1), the structure modality yields slightly worse or similar performance, while the overall performance of the text and Interpro modalities lags further behind.

To investigate if the individual modalities are complementary, we evaluated the performance of the consensus approach of simply averaging the predictions of the four modalities (sequence, structure, text, and Interpro domain) on the Test_Zero dataset (see the results in Table S9, available as supplementary data at *Bioinformatics* online). The consensus approach, achieves superior performance across all ontologies, exceeding that of both the central sequence modality and each individual non-central modality. The results highlight the efficacy of multimodal integration in enhancing the robustness and accuracy of zero-shot protein function prediction. Detailed results and examples, including alignment visualizations and additional zero-shot evaluations, are presented in the Supplementary Note S1, available as supplementary data at *Bioinformatics* online.

To further assess the semantic meaning of our zero-shot predictions, we analyze the Information Content (IC) of correctly predicted zero-shot GO terms, which reflects their level of specificity in the Gene Ontology hierarchy. This analysis allows us to determine whether FunBind primarily predicts generic, high-level GO terms or is capable of identifying more specific functional concepts. The results, presented in Supplementary Note S4, available as supplementary data at *Bioinformatics* online, show that correctly predicted zero-shot GO terms span a broad range of IC values, including a substantial proportion of high-IC terms. This indicates that FunBind is not limited to generic annotations but can capture semantically specific protein functions in the zero-shot setting. Furthermore, the IC distribution of correct predictions closely matches that of the ground-truth annotations, suggesting that the model does not exhibit a bias toward low-IC (generic) terms. Overall, these findings demonstrate that the learned multimodal representation enables meaningful semantic generalization beyond the training labels.

We also conduct an ablation study to evaluate the performance of our zero-shot framework against three baseline models: E5 Wang *et al*. (2024), BioGPT Luo *et al*. (2022), and LLaMA Touvron *et al*. (2023). Specifically, we compare retrieval performance across ontologies using the Text and InterPro modalities that use textual descriptions, enabling a systematic analysis of how embedding geometry, domain-specific knowledge, and general-purpose language modeling affect zero-shot protein function prediction. These results are shown in Supplementary Note S5, available as supplementary data at *Bioinformatics* online. Overall, FunBind achieves the best and most consistent performance on average among the four methods across function ontologies. E5 provides the strongest baseline performance and BioGPT outperforms LLaMA due to domain-specific knowledge.

We further evaluate this ablation setting under a sequence redundancy reduction constraint (<30% identity). The results, shown in Supplementary Note S6, available as supplementary data at *Bioinformatics* online, demonstrate that FunBind maintains strong performance under this stricter setting. Importantly, the relative trends across modalities and baselines remain consistent, indicating that the performance gains of FunBind are not solely driven by sequence similarity. These findings demonstrate that FunBind captures functional signals that generalize beyond homologous relationships, supporting its applicability in realistic, low-similarity scenarios.

Finally, removing the contrastive objective leads to a consistent performance drop across all ontologies (Fig. S17, available as supplementary data at *Bioinformatics* online), demonstrating that contrastive learning is critical for multimodal alignment and zero-shot generalization.

### 3.3 Comparison of fine-tuned FunBind and existing function prediction methods

As shown in (Fig. 1), we also fine-tuned the pretrained FunBind on the training dataset via supervised learning to predict protein function and compared it with five existing methods on the Test_All dataset. Test_All comprises the new proteins released after the latest release date of the proteins in the training data (see “Methods” section for details).

Specifically, the fine-tuned FunBind was compared with two traditional baseline methods (Naive, DiamondBlast(Boadu and Cheng 2024)) and three current state-of-the-art (SOTA) deep learning methods (TransFew(Boadu and Cheng 2024), SPROF-GO(Yuan *et al*. 2023) and DeepGO-SE (Kulmanov *et al*. 2024)), in terms of multiple metrics, including F${\;}_{\max}$, Area under the Precision-Recall curve (AUPR), weighted F${\;}_{\max}$, and S${\;}_{\min}$ of measuring the uncertain/missing information in function predictions (Zhou *et al*. 2019, Piovesan *et al*. 2024) (See the detailed definition of the evaluation metrics and a summary of the five function prediction methods in Supplementary Notes S2 and S3, available as supplementary data at *Bioinformatics* online respectively). FunBind produces its multimodal consensus predictions by simply averaging the predictions of the function classification head of available non-function modalities (Sequence, Text, InterPro, and Structure). The prediction performance of each individual modality is also reported.

For SPROF-GO, we obtained its predictions from its web server (as of 9 February 2025), while for DeepGOSE, TransFew, Naive and DiamondBlast, we generated their predictions by running them locally. FundBind, TransFew, SPROF-GO, DeepGO-SE, Naive, and the Sequence modality made predictions for all the proteins (coverage = 1), while the other individual modalities made predictions for 90% to 98% of proteins because some modalities are missing for a small portion of proteins. DiamondBlast only made predictions for 75% to 78% of proteins in three function categories because it could not found homologous hits for some proteins.

The results of FunBind, the individual modalities of FunBind (four single-modal models), and the five existing methods on the Test_All dataset are presented in Table 2. The Precision-Recall curves of the methods are shown in Fig. S13, available as supplementary data at *Bioinformatics* online.

**Table 2:** Performance of multimodal FunBind, five existing methods, and four single-modal models on Test_All dataset.

Methods		$F_{\max}$ (↑)			$WF_{\max}$ (↑)			*AUPR* (↑)			$S_{\min}$ (↓)			Coverage		
		CC	MF	BP	CC	MF	BP	CC	MF	BP	CC	MF	BP	CC	MF	BP
*Baseline*	Naive	0.588	0.534	0.267	0.323	0.372	0.183	0.483	0.282	0.144	6.351	5.034	17.532	1.0	1.0	1.0
	DiamondBLAST	0.587	0.563	0.347	0.470	0.480	0.292	0.050	0.047	0.043	7.566	5.146	23.342	0.78	0.76	0.75
*SOTA*	DeepGO-SE	0.681	0.634	0.402	0.502	0.519	0.323	0.667	0.552	0.320	5.481	4.020	16.085	1.0	1.0	1.0
	SPROF-GO	*0.718*	*0.680*	0.414	*0.579*	0.577	0.334	**0.747**	*0.619*	0.329	*4.922*	*3.518*	15.476	1.0	1.0	1.0
	TransFew	0.703	*0.680*	0.421	0.570	*0.593*	0.349	0.631	0.528	0.283	5.182	3.735	16.173	1.0	1.0	1.0
*Single-modal*	Structure	0.671	0.648	0.413	0.526	0.563	0.335	0.659	0.546	0.299	5.352	3.801	15.660	0.91	0.90	0.90
	Interpro	0.657	0.643	0.421	0.505	0.546	0.344	0.668	0.552	0.313	5.862	3.977	15.670	0.95	0.94	0.93
	Sequence	0.695	0.663	*0.449*	0.557	0.570	*0.375*	0.516	0.491	0.330	5.413	3.853	15.472	1.0	1.0	1.0
	Text	0.692	0.677	0.441	0.546	0.586	0.366	0.713	0.585	*0.334*	5.339	3.691	*15.145*	0.98	0.98	0.98
	FunBind	**0.724**	**0.699**	**0.475**	**0.596**	**0.611**	**0.404**	*0.745*	**0.637**	**0.389**	**4.824**	**3.474**	**14.626**	1.0	1.0	1.0

Bold font highlights the best performance and underline denotes the second best performance.

The results show that FunBind outperforms all the existing methods and four individual modalities, in terms of almost all the metrics for all three function categories (CC, MF, and BP). It is clearly demonstrated that integrating multiple modalities with FundBind advances the state of the art of protein function prediction.

FundBind not only consistently outperform three state-of-the-art deep learning methods (TransFew (Boadu and Cheng 2024), SPROF-GO (Yuan *et al*. 2023) and DeepGO-SE (Kulmanov *et al*. 2024)), but also improves the prediction accuracy by a pronounced margin in some situations, particularly for BP. For instance, the AUPR of FundBind for BP is 0.389, more than 6 percentage points higher than 0.320, 0.329, 0.283 of DeepGO-SE, SPROF-GO and TransFew.

Among the four individual modalities, Sequence achieves the best overall performance on its respective test set, closely followed by Text, while Structure and InterPro exhibit comparable but slightly lower performance. Notably, all four individual modalities substantially outperform the two baseline methods (Naive and DiamondBLAST). Furthermore, the scores achieved by Sequence and Text are comparable to those of the three deep learning methods. However, it is important to note that this comparison is not entirely fair, as some of the methods do not make predictions for all proteins (i.e., coverage < 1), potentially biasing the results.

To further evaluate the robustness and generalization of FunBind and the other methods, we assessed their performance on the more stringent Test_Novel dataset, which includes only proteins with less than 30% sequence identity to any protein in the training set. This dataset focuses on proteins that are dissimilar to the training data, providing a rigorous test of a model’s ability to generalize beyond similar sequences (see results in Table S10 and Fig. S14, available as supplementary data at *Bioinformatics* online).

On this challenging benchmark, FunBind consistently ranks either first or second in terms of all metrics across all function categories, yielding the best overall performance, followed by SPROF-GO. In particular, like its performance on Test_All, it achieves the highest scores in BP across all metrics. Its scores in BP are substantially higher than the three deep learning methods (SPROF-GO, TransFew, and DeepGO-SE) as well as the two traditional methods, showcasing its strength in predicting biological processes.

Importantly, FunBind’s performance on Test_Novel remains stable and comparable to its performance on the broader Test_All dataset, underscoring it generalizes well to new protein sequences that are very dissimilar to the training proteins. Moreover, The superior performance of FunBind over each individual modality highlights its ability to extract and integrate complementary signals from diverse modalities to improve protein function prediction.

We also conduct an ablation study to systematically evaluate the contribution of different modality alignments through pairwise modality alignments/combinations. The results in Table S11, available as supplementary data at *Bioinformatics* online show that pairwise modality combinations consistently outperform single-modality models across all evaluation metrics, demonstrating the benefit of integrating complementary information from two different modalities. Pairwise combinations involving the sequence modality (e.g., sequence–text and sequence–InterPro) achieve the best performance among all pairwise settings, indicating that sequence serves as a strong backbone for multimodal integration. Notably, non-sequence pairs (e.g., structure–text and structure–InterPro) also yield competitive results, suggesting that meaningful functional signals can be captured even without direct sequence input. Overall, the full multimodal FunBind model consistently achieves the best performance across all metrics and ontologies, confirming that integrating all modalities provides the most comprehensive and effective representation for protein function prediction.

## 4 Discussion

Our results demonstrate that the multimodal model (FunBind) combining sequence, structure, textual descriptions, and InterPro domain annotations can enable accurate unsupervised zero-shot protein function prediction across all three GO function categories and further improve supervised protein function classification over the current state-of-the-art methods. The analysis reveals distinct contributions from individual modalities and their complementarity. Combining them consistently improves prediction accuracy over each individual modality.

Despite these promising findings, several avenues exist for future improvement. First, rather than relying on frozen encoders for initial representation generation, fine-tuning the base encoders, such as ESM for sequences, ProtsT5 for structure, and LLaMA for text, may further improve performance by adapting them to the function prediction task. Even selectively fine-tuning some of them might offer some gains without the full cost of joint training.

Second, the structure modality may be underutilized in our current implementation. The 3Di representation may be too coarse and lose some structural information. A more detailed representation of protein structures, such as graph-based representations and atomic-level embeddings, may provide more robust structural signals that contribute more to function prediction. A more advanced structure encoder that directly generates structural embeddings from a 3D protein structure may further enhance function prediction.

Third, the multimodal model of FunBind could be extended to incorporate additional modalities that capture orthogonal biological information. One promising direction is the integration of protein–protein interaction (PPI) networks (Wang *et al*. 2025), which can encode relational context and functional associations often missed by individual protein-level descriptors. Combining PPI data with sequence, structure, domain annotations and text could unlock new capabilities, especially for inferring biological processes that depend on protein-protein interactions a lot.

Finally, in addition to protein function prediction, we envision FunBind can be used to generate the unique, unified representations of protein sequences, structures, text description, and Interpro domain annotations imbued with protein function contexts for various other downstream protein bioinformatics tasks, such as protein interaction prediction and protein design.

## Supplementary Material

btag356_Supplementary_Data
