# Supplementary material for: A unified multimodal model for generalizable zero-shot and supervised protein function prediction
Source: Bioinformatics. 2026 Jun 4;42(6):btag356. doi: 10.1093/bioinformatics/btag356 (PMC13281926; doi:10.1093/bioinformatics/btag356)
Supplement: btag356_Supplementary_Data [file btag356_supplementary_data.pdf]

FunBind

A unified multimodal model for generalizable zero-shot and supervised protein function prediction

Supplementary Materials

Author: Frimpong Boadu, Yanli Wang, Jianlin Cheng

Table S1: Statistics of Training Data. The number of proteins for each of four non-function modalities (Sequence, Structure, Text, and Interpro domain annotations) in each of the three gene ontology (GO) function categories (CC: cellular component, MF: molecular function, and BP: biological process) is reported. The total number of unique proteins for each modality spanning all three function categories is listed in the "Pretraining" column.

Ontology	Sequence	Structure	Text	InterPro
CC	92912	85491	75625	85722
MF	78637	72406	68500	74422
BP	92210	80243	74519	82370
Pretraining	133339	125021	110425	126861

Table S2: Statistics of Test_All Dataset

Ontology	Sequence	Structure	Text	InterPro
CC	1883	1728	1661	1799
MF	2441	2217	2170	2315
BP	2585	2347	2238	2424

Table S3: Statistics of Test_Novel Dataset

Ontology	Sequence	Structure	Text	InterPro
CC	206	169	201	159
MF	231	179	219	183
BP	282	213	269	209

Table S4: Statistics of Test_Zero dataset for zero-shot prediction. The number of proteins having each of four modalities for each function ontology / category (CC: cellular component, MF: molecular function, and BP: biological process) is reported. The Intersection column lists the number of proteins having all four non-function modalities. The All row lists the sum of the numbers of proteins in CC, MF and BP combined.

Ontology	Sequence	Structure	Text	InterPro	Intersection
CC	73	59	73	69	56
MF	192	43	190	186	43
BP	427	92	419	402	86
All	659	185	650	625	176

Table S5: Comparison of ESM2 and ProstT5 as sequence encoders for the supervised classification task. Evaluation is based on F_{max}, WF_{max}, AUPR, and S_{min} across the three GO ontologies: Cellular Component (CC), Molecular Function (MF), and Biological Process (BP). Overall, both encoders perform similarly, with ESM2 showing slightly stronger results in text and InterPro integration, while ProtsT5 performs marginally better with structural embeddings.

**Methods**		F_{max} (↑)			WF_{max} (↑)			$AUPR$ (↑)			S_{min} (↓)		
		**CC**	**MF**	**BP**	**CC**	**MF**	**BP**	**CC**	**MF**	**BP**	**CC**	**MF**	**BP**
**Sequence**	ESM2	0.696	0.663	0.449	0.557	0.571	0.376	0.517	0.492	0.330	5.414	3.853	15.472
	ProstT5	0.705	0.676	0.444	0.565	0.585	0.368	0.620	0.587	0.331	5.106	3.710	15.391
**Structure**	ESM2	0.672	0.649	0.413	0.527	0.563	0.335	0.659	0.546	0.299	5.352	3.801	15.660
	ProstT5	0.672	0.648	0.411	0.526	0.558	0.333	0.664	0.541	0.2975	5.357	3.814	15.656
**Text**	ESM2	0.692	0.677	0.441	0.546	0.586	0.366	0.713	0.585	0.334	5.340	5.862	15.144
	ProstT5	0.695	0.676	0.437	0.550	0.583	0.362	0.707	0.574	0.328	5.273	5.764	15.289
**Interpro**	ESM2	0.657	0.643	0.421	0.505	0.546	0.344	0.668	0.552	0.313	3.977	3.691	15.670
	ProstT5	0.663	0.640	0.418	0.513	0.537	0.339	0.657	0.553	0.311	3.938	3.694	15.801
**FunBind**	ESM2	0.724	0.699	0.475	0.596	0.611	0.404	0.745	0.637	0.389	4.824	3.474	14.625
	ProstT5	0.727	0.698	0.467	0.594	0.611	0.393	0.743	0.642	0.381	4.731	3.487	14.669

Table S6: Summary of textual descriptions on the training Data. 110425 proteins in the training data have some textual information. The number of proteins for each text field is reported.

**Training Data**								
	**Name**	**Desc.**	**Func.**	**Subunit**	**Sub. Loc.**	**Induc.**	**Tiss. Spec.**	**Sim.**
# of proteins	110425	110425	81618	53990	90246	12773	31163	88831
Coverage	100%	100%	73.9%	48.9%	81.7%	11.6%	28.2%	80.4%
**Test Data**								
	**Name**	**Desc.**	**Func.**	**Subunit**	**Sub. Loc.**	**Induc.**	**Tiss. Spec.**	**Sim.**
# of proteins	4952	4952	3506	3531	2161	3626	1046	469
Coverage	98.7%	98.7%	69.9%	70.4%	43.1%	72.3%	20.8%	9.3%

Table S7: Effect of removing individual text components on protein function prediction performance, evaluated across Cellular Component (CC), Molecular Function (MF), and Biological Process (BP). Each row shows performance when one specific text field is excluded, while all others are retained. The complete model with all components included is shown in the last row for reference. The **Function** field has the most significant overall impact. Additionally, **Subcellular Location** is particularly important for CC, and **Similarity** contributes notably to MF. Both features also influence BP performance. In contrast, **Tissue Specificity** and **Induction** have the least effect, as their removal does not noticeably degrade performance, suggesting lower predictive utility in this setting.

Formatting legend: **Bold** indicates the best performance across all settings for a given metric. Underlined values represent the second-best. Red-shaded cells indicate the most severe performance degradation. Green-shaded rows mark cases where removal had little or no negative effect.

**Removed Text Field**	F_{max} (↑)			WF_{max} (↑)			$AUPR$ (↑)			S_{min} (↓)		
	**CC**	**MF**	**BP**	**CC**	**MF**	**BP**	**CC**	**MF**	**BP**	**CC**	**MF**	**BP**
No Function	0.596	0.582	0.372	0.479	0.501	0.313	0.5227	0.4374	0.2422	6.308	4.312	16.232
No Induction	**0.604**	**0.605**	**0.393**	0.491	**0.530**	**0.333**	0.5302	**0.4573**	0.2627	**6.184**	**4.093**	15.792
No Subcellular Location	0.580	0.604	0.384	0.454	0.528	0.324	0.5170	0.4565	0.2579	6.491	4.118	15.899
No Similarity	0.601	0.583	0.387	0.490	0.502	0.325	0.5195	0.4422	0.2583	6.220	4.287	15.807
No Subunit	0.603	0.600	**0.393**	0.491	0.524	0.332	**0.5478**	0.4530	**0.2639**	6.185	4.111	**15.743**
No Tissue Specificity	**0.604**	0.603	0.391	**0.494**	0.528	0.332	0.5319	0.4562	0.2638	**6.187**	4.104	15.750
All	0.606	0.604	0.392	0.494	0.530	0.333	0.530	0.456	0.263	6.157	4.101	15.759

Note: Only proteins with annotations across all modalities were included. Dataset sizes—BP: 1895, CC: 1395, MF: 1850. Missing counts for each modality: Function (4466), Location (4339), Similarity (4158), Induction (4511), Subunit (4501), Tissue Specificity (4502).

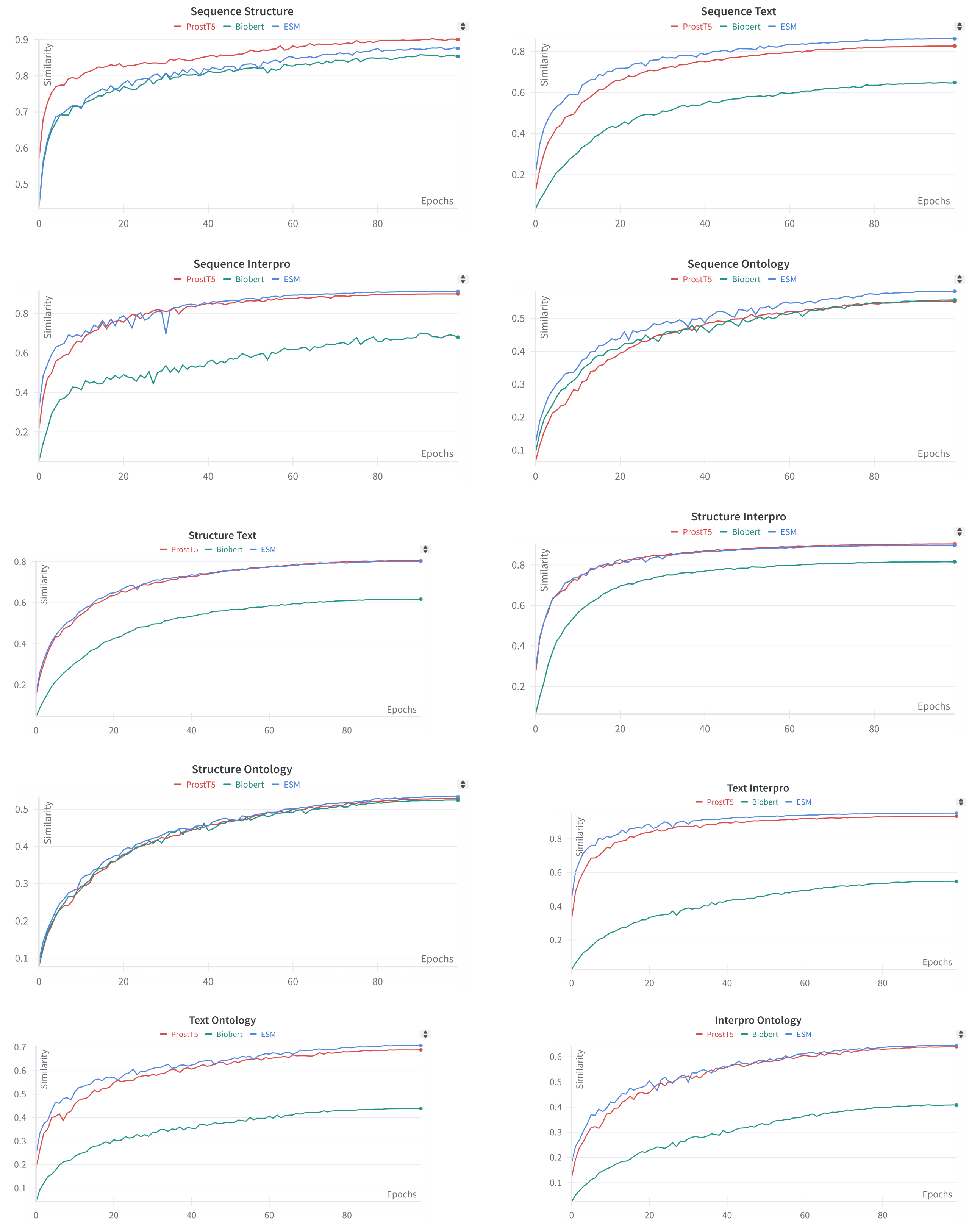

Figure S1: Cross-modal similarity on the validation set during pretraining, illustrating the alignment between modality embeddings across epochs. Each plot shows the cosine similarity between pairs of modality embeddings. When ”ProstT5” indicates that ProstT5 was used as the sequence encoder; similarly, ”BioBERT” indicates the use of BioBERT as the base encoder for Text and Interpro. In all other cases, the default encoders are: ESM for sequence, ProstT5 for structure, and LLaMA2 for text, InterPro, and ontology.

Example Protein -- Q9UJZ1

Sequence: MLARAARGTGALLLRGSLLASGRAPRRASSGLPRNTVVLFVPQQEAWVVERMGR **...** KMS

Structure: dddddddddddddddddddddddpddpppppfdaqdqkdaaapqkwwfkdfpnh **...** pvd

Textual Annotation: PROTEIN NAME: STML2_HUMAN; **DESCRIPTION**: Stomatin-like protein 2, mitochondrial; **FUNCTION**: Mitochondrial protein that probably regulates the biogenesis and the activity of mitochondria. Stimulates cardiolipin biosynthesis, binds cardiolipin-enriched membranes where it recruits and stabilizes some proteins including prohibitin and may therefore act in the organization of functional microdomains in mitochondrial membranes. Through regulation of the mitochondrial function may play a role into several biological processes including cell migration, cell proliferation, T-cell activation, calcium homeostasis and cellular response to stress. May play a role in calcium homeostasis through negative regulation of calcium efflux from mitochondria. Required for mitochondrial hyperfusion a pro-survival cellular response to stress which results in increased ATP production by mitochondria. May also regulate the organization of functional domains at the plasma membrane and play a role in T-cell activation through association with the T- cell receptor signaling complex and its regulation.; **SUBUNIT**: Forms homooligomers. Interacts with MFN2; may form heterooligomers. Interacts with CACNA2D2 (By similarity). Interacts with PHB1 and PHB2; recruits them to cardiolipin-enriched mitochondrial membranes and stabilizes them.; **SUBCELLULAR LOCATION**: Cell membrane ; Peripheral membrane protein . Mitochondrion . Mitochondrion inner membrane ; Lipid-anchor . Mitochondrion intermembrane space . Membrane raft . Cytoplasm, cytoskeleton . Note=Behaves as an integral membrane protein of the mitochondrion despite the absence of a detectable transmembrane domain . Also associates with the actin cytoskeleton and membrane rafts in activated T-cells (PubMed:10713127, PubMed:18641330). A minor pool is associated with the plasma membrane and is enriched at the immunological synapse in activated T-cells.; **INDUCTION**: Up-regulated in activated B- and T-cells and upon mitochondrial stress by chloramphenicol.; **TISSUE SPECIFICITY**: Ubiquitously expressed at low levels. Expressed in lymphoid tissues (at protein level).; **SIMILARITY**: Belongs to the band 7/mec-2 family.

Interpro: Protein Name: STML2_HUMAN; **Description**: Stomatin-like protein 2, mitochondrial; **Interpro Name**: Band 7/SPFH domain superfamily, **Interpro Type**: Homologous_superfamily; **Interpro Name**: STML2-like, C-terminal extension, **Interpro Type**: Domain; **Interpro Name**: Band 7/mec-2 domain-containing protein, **Interpro Type**: Family; **Interpro Name**: Stomatin/HflK family, **Interpro Type**: Family; **Interpro Name**: Band 7 domain, **Interpro Type**: Domain

--**zeroshot**--

Ontology: Cellular Component:: mitochondrial inner membrane: The inner, i.e. lumen-facing, lipid bilayer of the mitochondrial envelope. It is highly folded to form cristae. ; mitochondrial intermembrane space: The region between the inner and outer lipid bilayers of the mitochondrial envelope. ; actin cytoskeleton: The part of the cytoskeleton (the internal framework of a cell) composed of actin and associated proteins. Includes actin cytoskeleton-associated complexes. ; membrane raft: Any of the small (10-200 nm), heterogeneous, highly dynamic, sterol- and sphingolipid-enriched membrane domains that compartmentalize cellular processes. Small rafts can sometimes be stabilized to form larger platforms through protein-protein and protein-lipid interactions. ; immunological synapse: An area of close contact between a lymphocyte (T-, B-, or natural killer cell) and a target cell formed through the clustering of particular signaling and adhesion molecules and their associated membrane rafts on both the lymphocyte and the target cell and facilitating activation of the lymphocyte, transfer of membrane from the target cell to the lymphocyte, and in some situations killing of the target cell through release of secretory granules and/or death-pathway ligand-receptor interaction. **Molecular Funtion::** cardiolipin binding: Binding to cardiolipin. ; T cell receptor binding: Binding to a T cell receptor, the antigen-recognizing receptor on the surface of T cells. ; GTPase binding: Binding to a GTPase, any enzyme that catalyzes the hydrolysis of GTP. **Biological Process::** T cell receptor signaling pathway: The series of molecular signals initiated by the cross-linking of an antigen receptor on a T cell. ; mitochondrion organization: A process that is carried out at the cellular level which results in the assembly, arrangement of constituent parts, or disassembly of a mitochondrion; includes mitochondrial morphogenesis and distribution, and replication of the mitochondrial genome as well as synthesis of new mitochondrial components. ; intracellular calcium ion homeostasis: A homeostatic process involved in the maintenance of a steady state level of calcium ions within a cell. ; protein complex oligomerization: The process of creating protein oligomers, compounds composed of a small number, usually between three and ten, of component monomers; protein oligomers may be composed of different or identical monomers. Oligomers may be formed by the polymerization of a number of monomers or the depolymerization of a large protein polymer.

Figure S2: A protein example with five modalities: sequence, structure, text description, InterPro domain annotations, and Gene Ontology terms

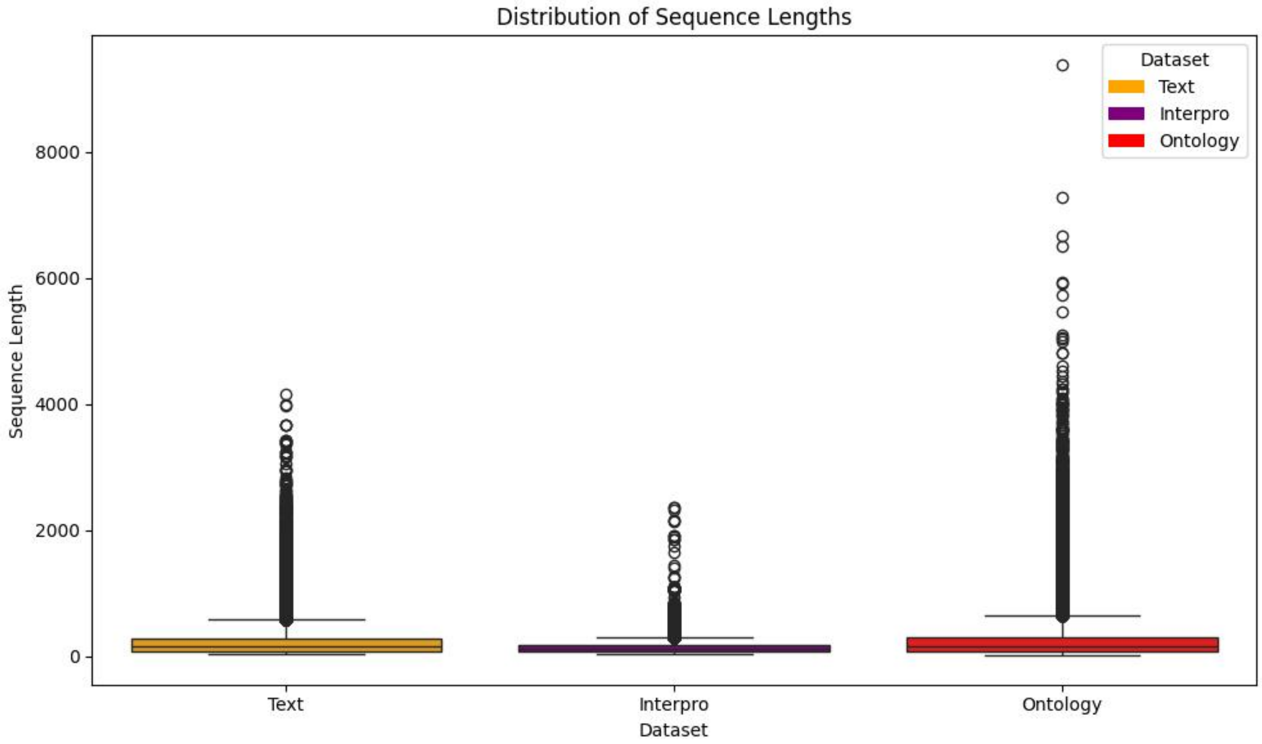

Figure S3: Distribution of text sequence lengths of three modalities: Text description, InterPro annotations, and Gene Ontology terms.

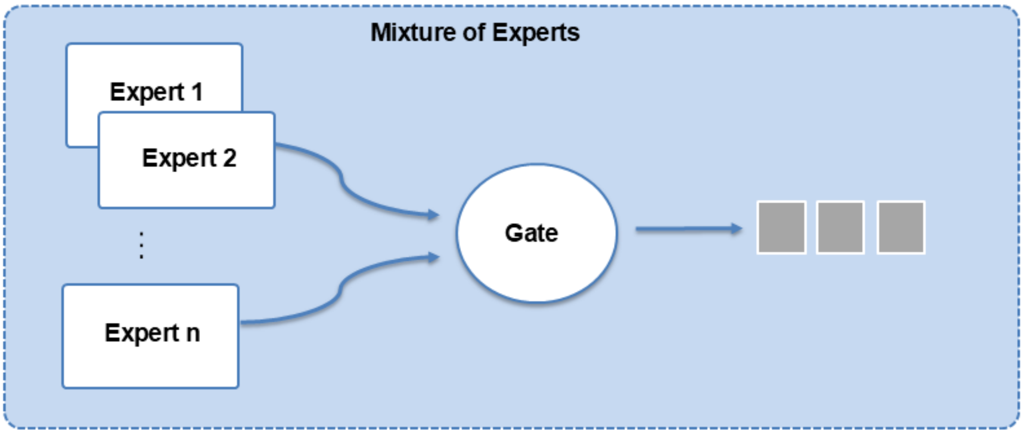

Figure S4: Illustration of the Mixture of Experts (MoE) architecture. Multiple expert networks process the input in parallel, and a gating mechanism dynamically weights their outputs to produce the final representation.

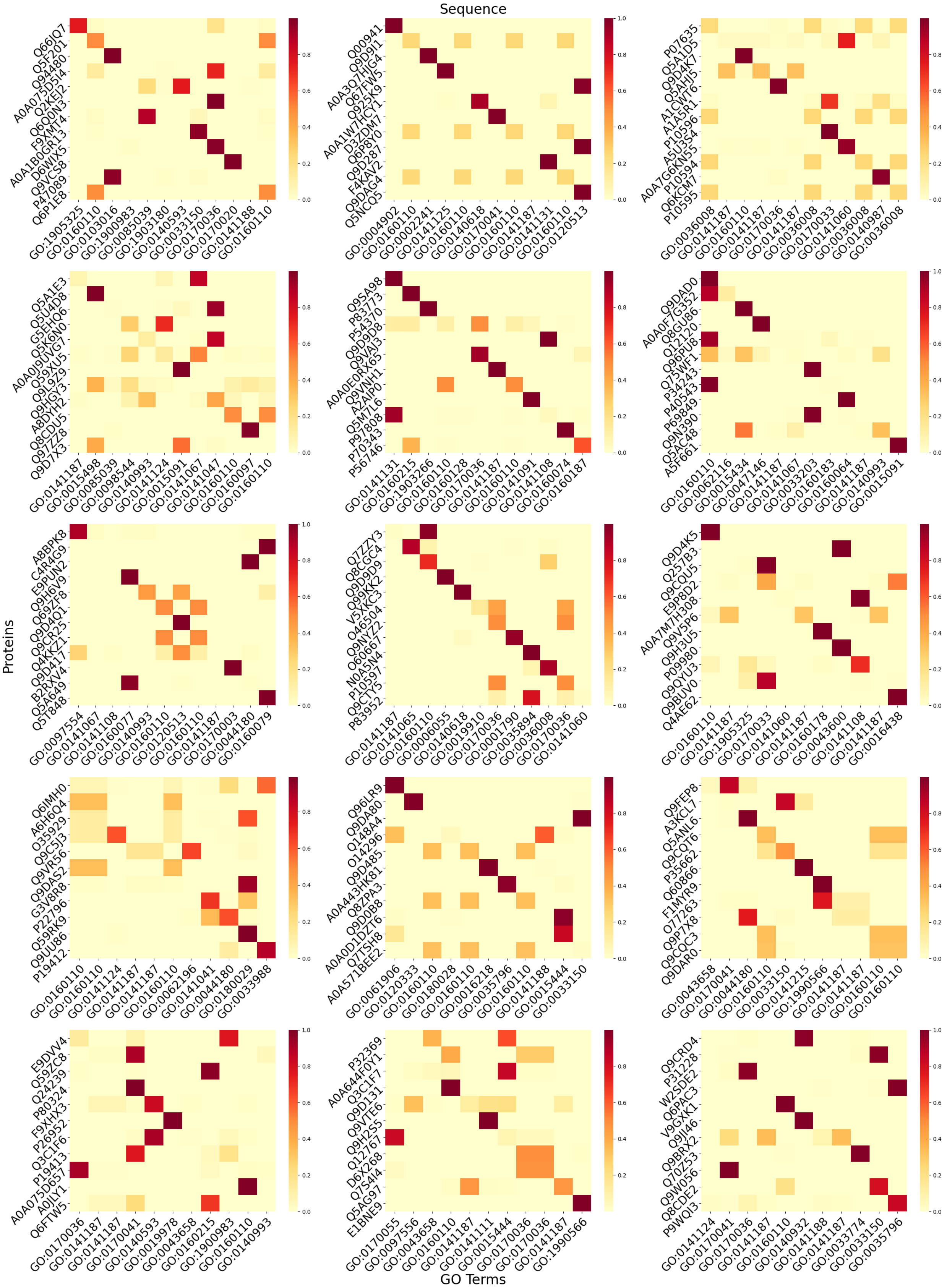

Figure S5: Heatmaps illustrating the similarity between proteins and GO terms for 12 groups, using the Sequence modality for retrieval. In each heatmap, rows correspond to proteins and columns to GO terms. The diagonal elements indicate the similarity between the embedding of the sequence modality of each protein and the embedding of its corresponding true GO term.

Table S8: Zero-shot prediction performance of FunBind using non-central modalities (Text, Interpro, and Structure). The results show that FunBind substantially improves zero-shot retrieval performance across all ontologies and modalities compared to the baseline using raw encoder embeddings. This improvement demonstrates that the multimodal alignment learned by FunBind effectively bridges modality-specific representation gaps, allowing functional information to be retrieved more accurately from non-central modalities such as text, InterPro domains, and structural features.

Ontology	Text				Interpro				Structure			
	**R@1**	**R@3**	**R@5**	**MRR**	**R@1**	**R@3**	**R@5**	**MRR**	**R@1**	**R@3**	**R@5**	**MRR**
**CC**	0.6559 (±0.0353)	0.8786 (±0.0214)	0.9624 (±0.0152)	0.7811 (±0.0195)	0.5827 (±0.0353)	0.8006 (±0.0178)	0.8412 (±0.0133)	0.7113 (±0.0211)	0.6694 (±0.0274)	0.7839 (±0.0093)	0.8179 (±0.0150)	0.7524 (±0.0184)
**MF**	0.6333 (±0.0415)	0.8429 (±0.0158)	0.8810 (±0.0213)	0.7476 (±0.0241)	0.6214 (±0.0516)	0.8381 (±0.0095)	0.8976 (±0.0214)	0.7445 (±0.0258)	0.6262 (±0.0533)	0.8643 (±0.0354)	0.9167 (±0.0160)	0.7546 (±0.0276)
**BP**	0.3883 (±0.0364)	0.6786 (±0.0250)	0.8083 (±0.0263)	0.5638 (±0.0209)	0.3464 (±0.0321)	0.6226 (±0.0261)	0.7595 (±0.0167)	0.5271 (±0.0191)	0.4619 (±0.0158)	0.6774 (±0.0202)	0.7679 (±0.0179)	0.6050 (±0.0089)
**All**	0.5490 (±0.0129)	0.7584 (±0.0192)	0.8496 (±0.0150)	0.6819 (±0.0102)	0.5067 (±0.0175)	0.7299 (±0.0122)	0.8279 (±0.0120)	0.6495 (±0.0126)	0.5714 (±0.0249)	0.7571 (±0.0147)	0.8420 (±0.0121)	0.6924 (±0.0142)

Note: Baseline represents retrieval using the modality encoders (LLaMA2 for ontology, text, and InterPro) without the FunBind multimodal alignment mechanism. In this setting, similarity is computed directly using the embeddings produced by the original encoders, without projecting them into the shared multimodal representation space learned by FunBind. In contrast, FunBind learns a contrastively aligned multimodal latent space that enables consistent representations across modalities.

Table S9: Mean and standard deviation of Recall@1, Recall@3, Recall@5, and MRR for consensus zero-shot function prediction on the Test_Zero dataset, averaged over 10 trials.

Ontology	R@1	R@3	R@5	MRR
**CC**	0.7850 (±0.0278)	0.9376 (±0.0222)	0.9661 (±0.0053)	0.8653 (±0.0168)
**MF**	0.6595 (±0.0427)	0.8595 (±0.0270)	0.9548 (±0.0128)	0.7708 (±0.0277)
**BP**	0.5024 (±0.0260)	0.7298 (±0.0131)	0.8238 (±0.0183)	0.6446 (±0.0162)
**All**	0.6429 (±0.0218)	0.8080 (±0.0125)	0.8861 (±0.0104)	0.7495 (±0.0151)

Supplementary Note S1: Additional zero-shot evaluations

Supplementary Figures S6, S7, and S8 visualize the alignment similarity between each non-central modality and the true GO terms, demonstrating that the modalities of many proteins are well aligned with their correct function annotations. Furthermore, **Supplementary Figure S9** illustrates the improved alignment achieved by the consensus approach, highlighting the benefits of integrating complementary modalities.

Finally, in addition to dividing the test proteins into small groups in the experiments above, we also used the entire set of test proteins as one single group (batch) for FunBind to perform zero-shot prediction. The results for the subset of proteins with data available for all four non-function modalities are reported in supplementary **Figure S10** and those for the entire protein set (regardless of modality availability) are reported in supplementary **Figure S11**. The zero-shot approach still predicts the novel terms of the proteins well in these settings, even though the accuracy is lower than dividing the proteins into smaller groups. The reason is that treating all the proteins as one single group requires retrieving true GO terms from more GO term candidates.

Supplementary Figure S12 illustrates four zero-shot prediction examples. Four proteins from the Text_Zero dataset (UniProt IDs: A8BPK8, P18335, Q12198, and Q64565) and their associated Gene Ontology (GO) terms were used to evaluate FundBind. The Sequence and Text modalities retrieved the correct GO term as top-1 prediction for three of the four proteins, while the Structure and InterPro modalities did so for two. For 14 out of 16 protein-modality combinations, there is at least one correctly predicted GO term in top 5 predictions. Some GO terms, such as L-amino acid metabolic process (GO:0170033) and non-proteinogenic amino acid metabolic process (GO:0170041) for protein Q12198, were consistently retrieved by all the modalities. Conversely, other correct terms were retrieved uniquely by specific modalities, underscoring modality-specific biases and the complementary nature of the different representations. For instance, for Q64565, the correct GO terms were retrieved as top-1 prediction by three different modalities (Sequence, Text and Structure), while Interpro failed to do so.

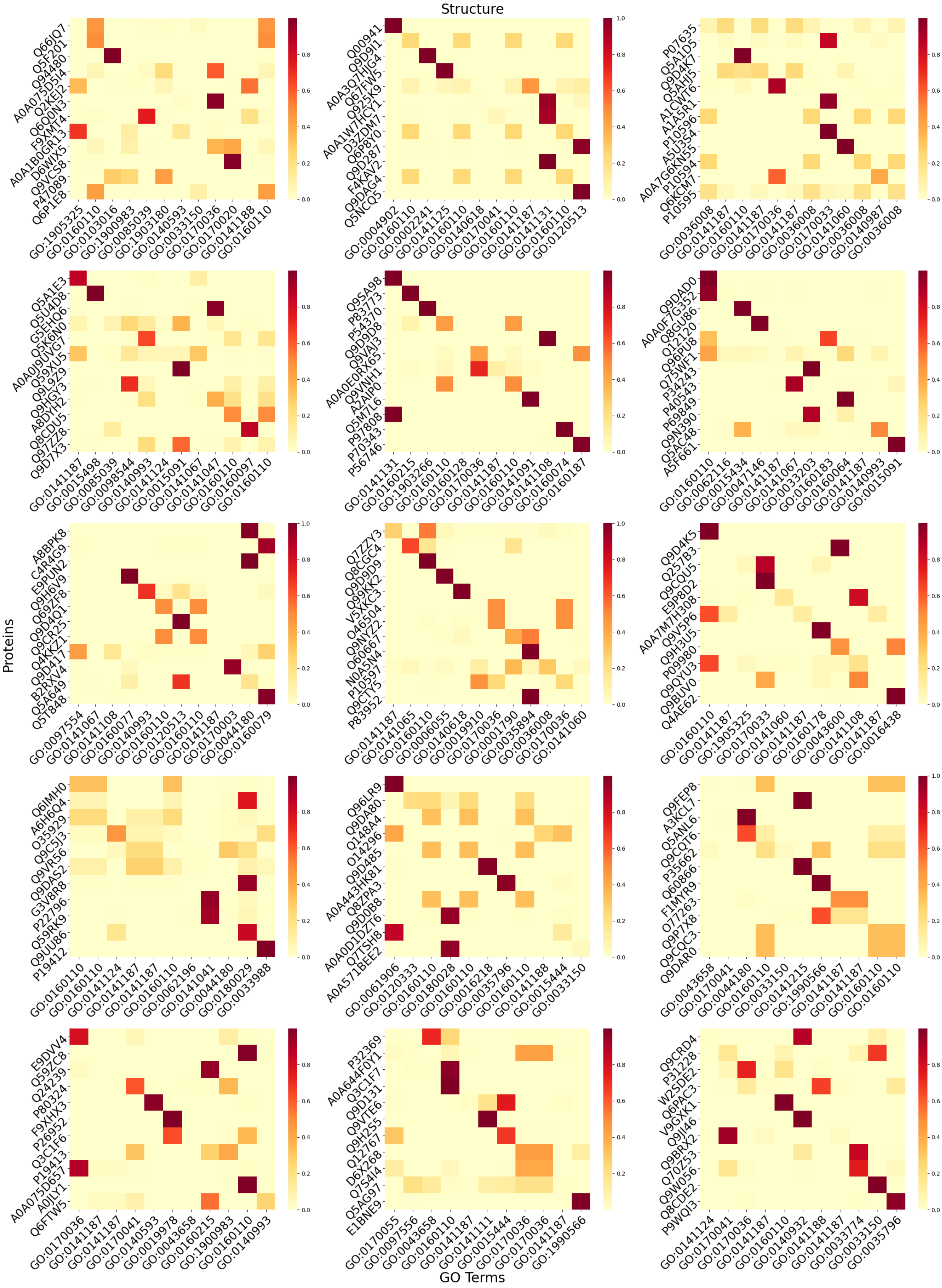

Figure S6: Heatmaps illustrating the similarity between proteins and GO terms for 12 groups, using Structure modality for retrieval. Each heatmap presents a similarity matrix, where rows correspond to proteins and columns to GO terms. The diagonal elements indicate the similarity between each protein and its true GO term.

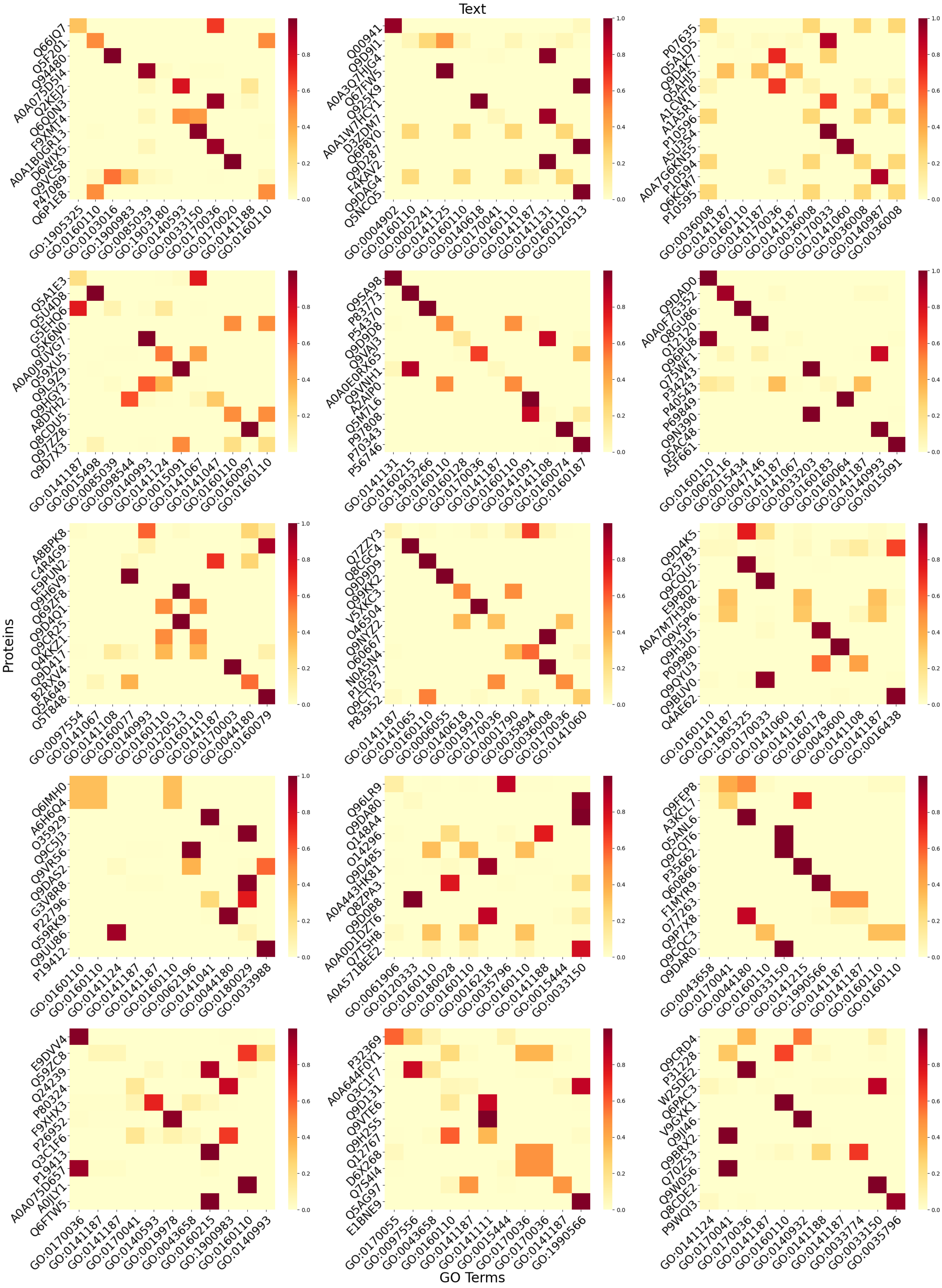

Figure S7: Heatmaps illustrating the similarity between proteins and GO terms for 12 groups, using Text modality for retrieval. Each heatmap presents a similarity matrix, where rows correspond to proteins and columns to GO terms. The diagonal elements indicate the similarity between each protein and its true GO term.

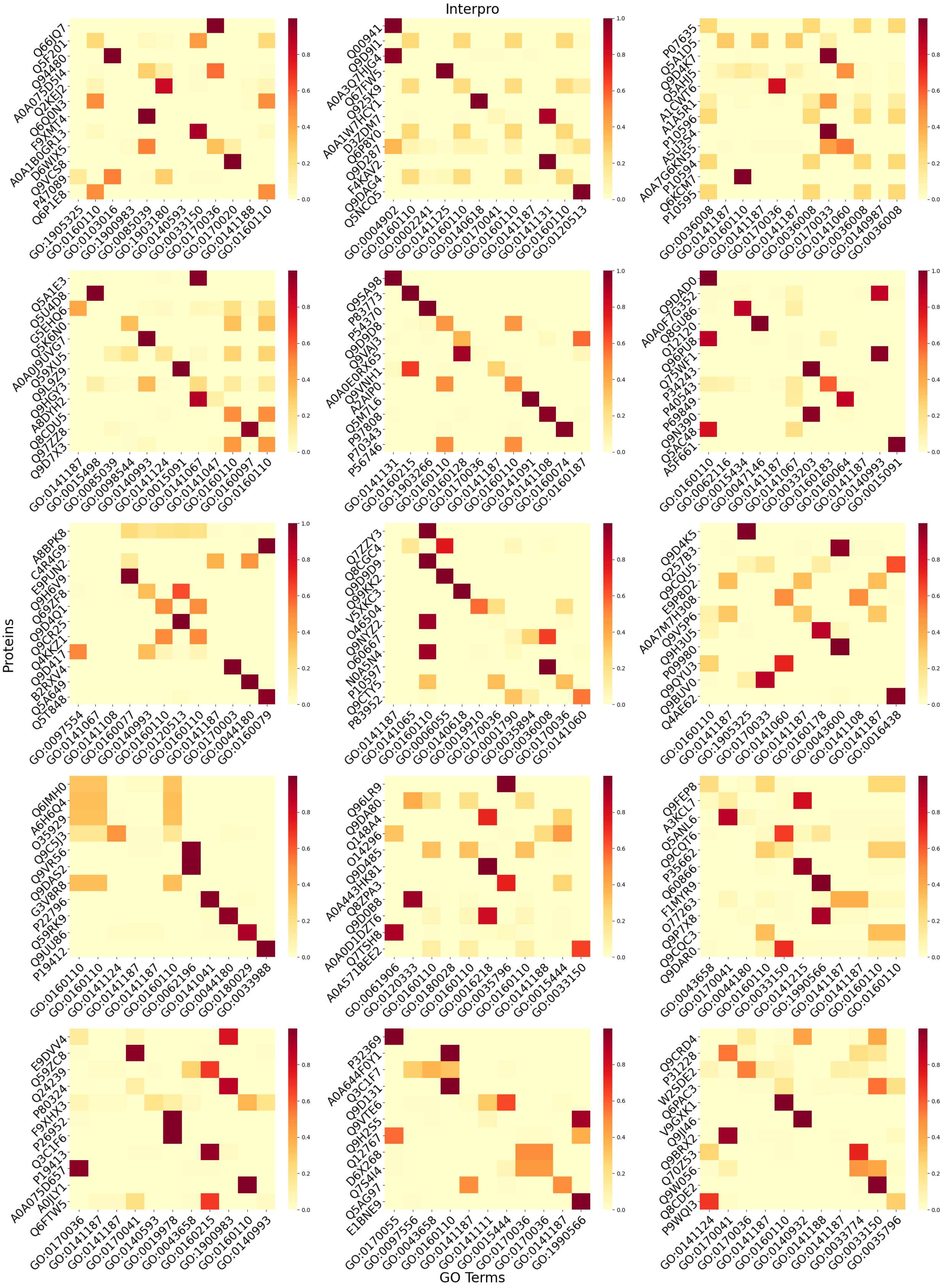

Figure S8: Heatmaps illustrating the similarity between proteins and GO terms for 12 groups, using Interpro modality for retrieval. Each heatmap presents a similarity matrix, where rows correspond to proteins and columns to GO terms. The diagonal elements indicate the similarity between each protein and its true GO term.

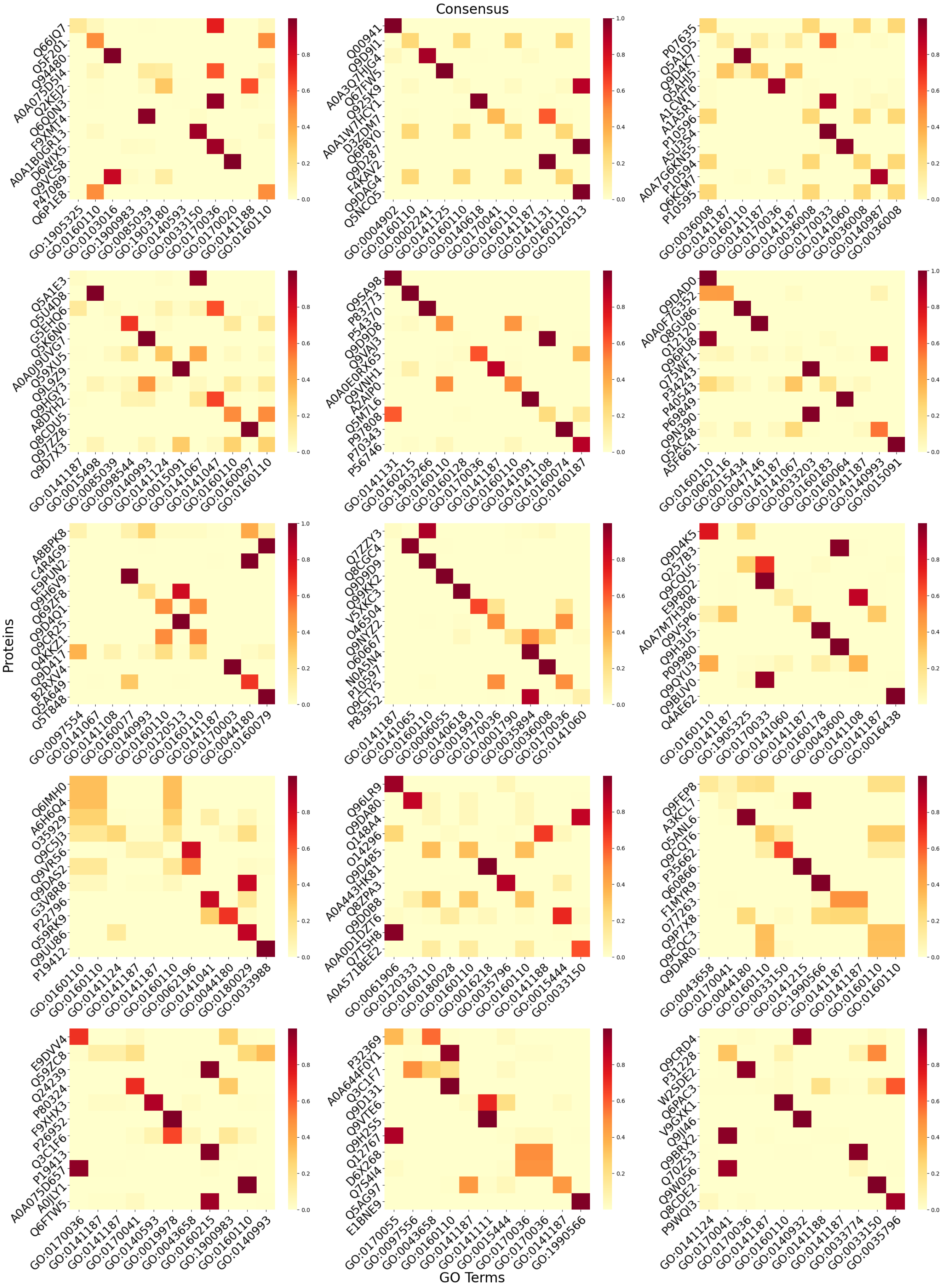

Figure S9: Heatmaps illustrating the similarity between proteins and GO terms, using the consensus predictions of all four modalities for retrieval. Each heatmap presents a similarity matrix, where rows correspond to proteins and columns to GO terms. The diagonal elements indicate the similarity between each protein and its true GO term.

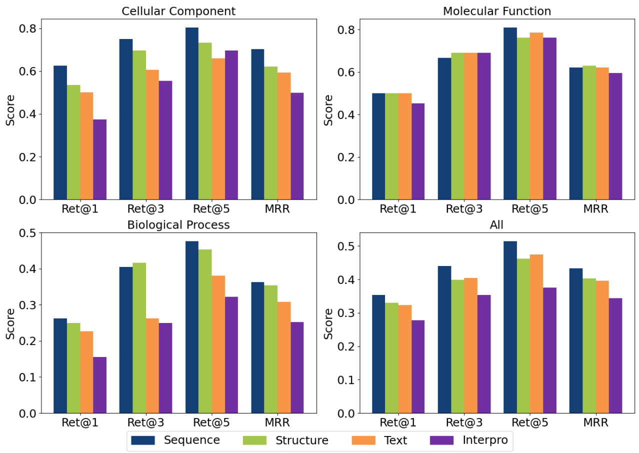

Figure S10: Zero-shot prediction performance for the test proteins with all modalities available in Test_zero dataset for each of the three GO function categories (Cellular Component, Molecular Function, Biological Process) and All function categories combined using individual modalities as queries. FunBind predict GO terms for all proteins in a single batch without dividing them into groups. The performance is reported using Ret@1, Ret@3, Ret@5, and MRR metrics. Each color represents a different modality: sequence, structure, text, and InterPro domain annotations.

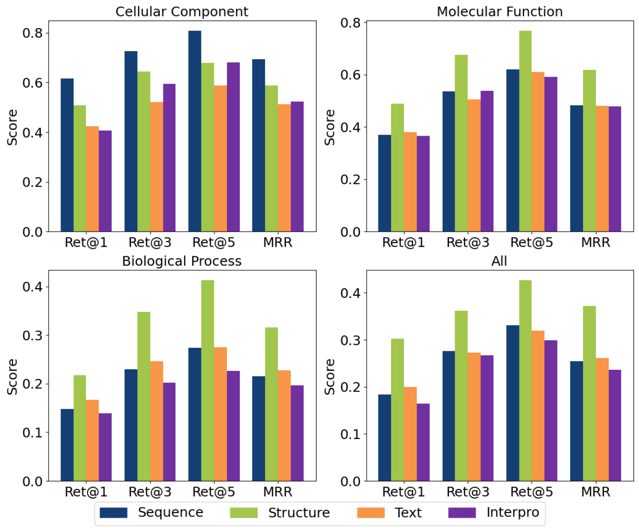

Figure S11: Zero-shot prediction performance on the entire test proteins (irrespective of modality availability) in the Test_Zero dataset for each of the three GO function categories (Cellular Component, Molecular Function, Biological Process) and all function categories combined using individual modalities as queries. FunBind predicts GO terms for all proteins in a single batch without dividing them into groups. The performance is reported using Ret@1, Ret@3, Ret@5, and MRR metrics.

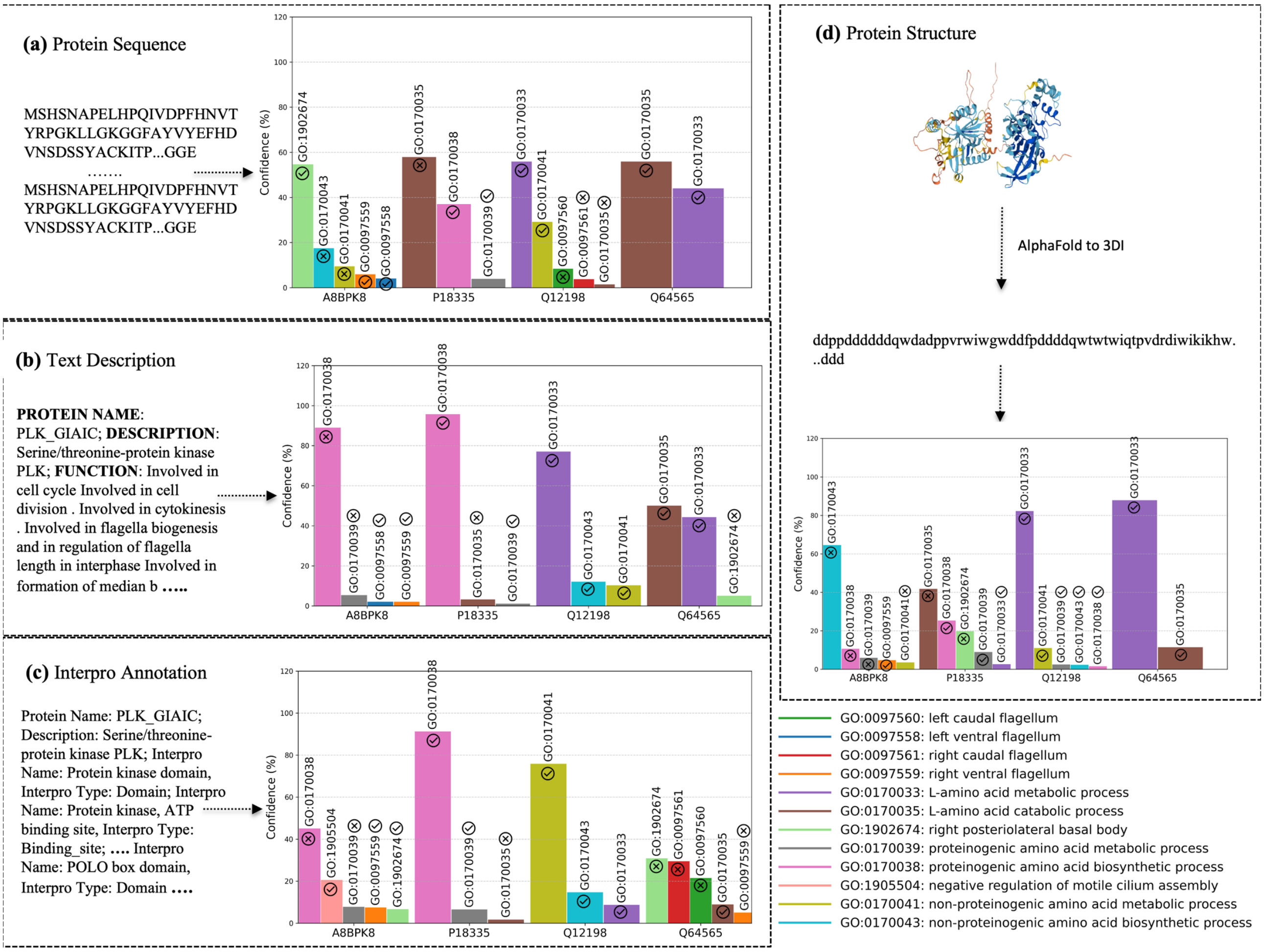

Figure S12: The zero-shot function predictions for four protein examples made by the four modalities of FunBind respectively. **(a)** protein sequence. **(b)** textual description. **(c)** InterPro domain annotation. **(d)** protein structure. The color-coded legends denote 12 different function terms. Two markers shown on top of each bar indicate if a predicted GO term is correct or not.

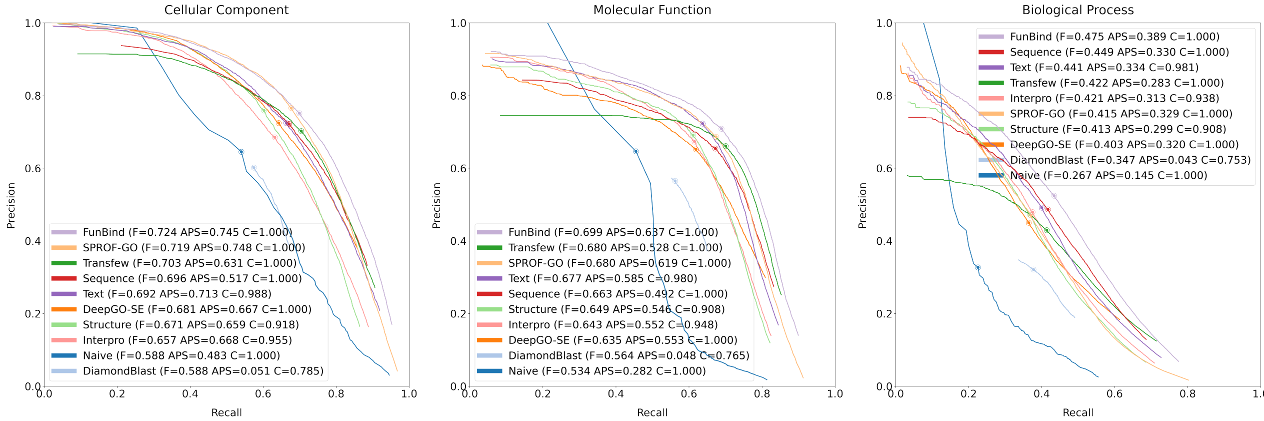

Figure S13: Precision-Recall curves of FunBind and existing methods for the three ontologies (BP, MF, and CC) on the Test_All dataset. The circled dot marks the point where each method achieves the highest F_{max}. Each plot also includes the AUPR score and the coverage of each method (the percent of proteins for which a method can make predictions). FunBind outperforms all existing methods, particularly leading in BP by a large margin.

Table S10: Performance of multimodal FunBind, five existing methods, and four single-modal models on Test_Novel dataset. Bold font highlights the best performance and underline denotes the second best performance.

	Methods	F_{max} (↑)			WF_{max} (↑)			$AUPR$ (↑)			S_{min} (↓)			Coverage		
		CC	MF	BP	CC	MF	BP	CC	MF	BP	CC	MF	BP	CC	MF	BP
**Baseline**	Naive	0.559	0.531	0.245	0.295	0.345	0.158	0.432	0.279	0.116	6.660	4.568	15.430	1.0	1.0	1.0
**Baseline**	DiamondBLAST	0.207	0.221	0.180	0.1659	0.208	0.163	0.007	0.014	0.010	9.483	5.685	16.034	0.16	0.20	0.19
**SOTA**	DeepGO-SE	0.672	0.608	0.376	0.472	0.464	0.319	0.663	0.514	0.292	5.510	3.977	14.202	1.0	1.0	1.0
**SOTA**	SPROF-GO	**0.719**	**0.662**	0.370	**0.572**	0.535	0.275	0.723	**0.588**	0.279	**4.729**	**3.458**	13.622	1.0	1.0	1.0
**SOTA**	TransFew	0.696	0.627	0.398	0.537	0.514	0.334	0.616	0.516	0.273	5.186	3.878	13.881	1.0	1.0	1.0
**Single-modal**	Structure	0.603	0.567	0.311	0.423	0.483	0.238	0.547	0.427	0.197	6.146	4.082	14.687	0.82	0.77	0.75
**Single-modal**	Interpro	0.549	0.543	0.374	0.402	0.449	0.317	0.481	0.387	0.244	6.882	4.226	13.674	0.77	0.79	0.74
**Single-modal**	Sequence	0.699	0.604	0.436	0.542	0.474	0.365	0.518	0.434	0.320	5.250	4.026	13.177	1.0	1.0	1.0
**Single-modal**	Text	0.630	0.613	0.401	0.431	0.489	0.317	0.600	0.460	0.285	6.205	3.988	13.435	0.97	0.94	0.95
	FunBind	0.718	0.656	**0.464**	0.569	**0.546**	**0.404**	**0.7330**	0.564	**0.380**	4.849	3.651	**12.563**	1.0	1.0	1.0

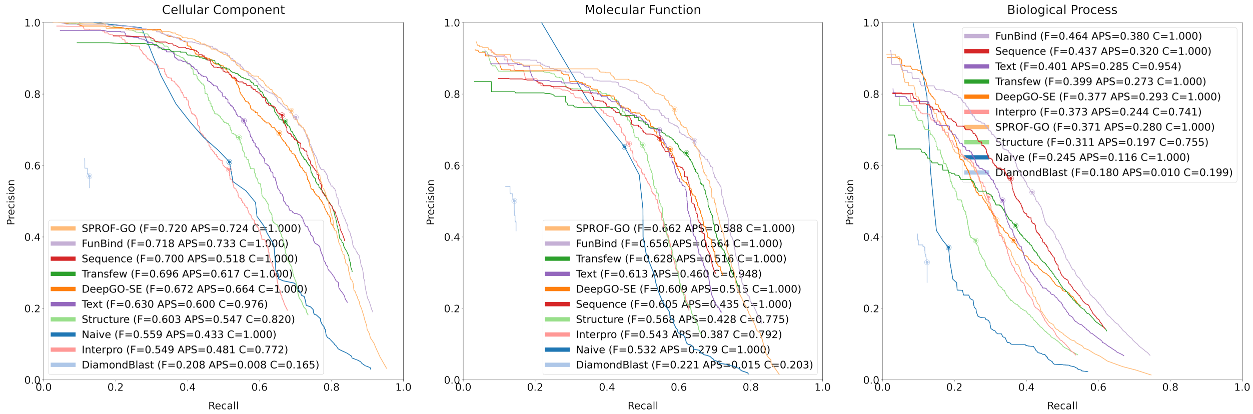

Figure S14: Precision-Recall curves of FunBind and existing methods for the three ontologies (BP, MF, and CC) on the Test_Novel dataset. The circled dot marks where each method achieves the highest F_{max}. Each plot also includes the AUPR score and coverage. FunBind outperforms all existing methods, particularly in BP.

Supplementary Note S2: Evaluation Metrics

In this work, we evaluate protein function prediction and zero-shot retrieval using three CAFA metrics—$F_{\max}$, $S_{\min}$, and weighted $F_{\max}$ [3, 10]. Additionally, we report the area under the precision-recall curve (AUPR) and standard retrieval metrics, including Recall@k (Ret@k) and Mean Reciprocal Rank (MRR). These metrics are defined as follows.

- **Precision**

$$\mathrm{pr}(\tau) = \frac{1}{m(\tau)} \sum_{i=1}^{m(\tau)} \frac{\sum_f \mathbb{I}(f \in P_i(\tau) \wedge f \in T_i)}{\sum_f \mathbb{I}(f \in P_i(\tau))}$$

- **Recall**

$$\mathrm{rc}(\tau) = \frac{1}{n_e} \sum_{i=1}^{n_e} \frac{\sum_f \mathbb{I}(f \in P_i(\tau) \wedge f \in T_i)}{\sum_f \mathbb{I}(f \in T_i)}$$

- **$\mathbf{F_1}$ Score**

$$F_{1(\tau)} = 2 \times \frac{\mathrm{pr}(\tau) \times \mathrm{rc}(\tau)}{\mathrm{pr}(\tau) + \mathrm{rc}(\tau)}$$

- **Maximum $\mathbf{F_1}$ Score**

$$F_{\max} = \max_{\tau} \left(F_1(\tau)\right)$$

  where f is a term, $P_i(\tau)$ is the set of predictions, T_i denotes the corresponding ground-truth, i represents the protein sequence under consideration, and τ is the decision threshold. $m(\tau)$ is the number of proteins sequences with at least one predicted score greater than or equal to the decision threshold τ, $\mathbb{I}(\cdot)$ is an indicator function, and n_e is the number of proteins in the test set for a particular test study.

- **Information Content** (ic) of term f is computed as:

$$\mathrm{IC}(f) = \log_2 \frac{1}{\Pr(f|P(f))}$$

- **Weighted precision**:

$$\mathrm{wpr}(\tau) = \frac{1}{m(\tau)} \sum_{i=1}^{m(\tau)} \frac{\sum_f ic(f) \cdot \mathbb{I}(f \in P_i(\tau) \wedge T_i(\tau))}{\sum_f ic(f) \cdot \mathbb{I}(f \in P_i(\tau))}$$

- **Weighted Recall**:

$$\text{wrc}(\tau) = \frac{1}{n_e} \sum_{i=1}^{n_e} \frac{\sum_f ic(f) \cdot \mathbb{I}(f \in P_i(\tau) \wedge T_i(\tau))}{\sum_f ic(f) \cdot \mathbb{I}(f \in T_i(\tau))}$$

Here, $\Pr(f|P(f))$ represents the probability that term f in the ontology is associated with a protein given that all of its parents are associated.

- **Remaining Uncertainty**

$$ru(\tau) = \frac{1}{ne} \sum_{i=1}^{ne} \sum_f ic(f) \cdot \mathbb{I}(f \notin P_i(\tau) \wedge f \in Ti)$$

- **Missing Information**

$$mi(\tau) = \frac{1}{ne} \sum_{i=1}^{ne} \sum_f ic(f) \cdot \mathbb{I}(f \in P_i(\tau) \wedge f \notin Ti)$$

- S_{min}

$$S_{min} = \min_{\tau} \sqrt{ru(\tau)^2 + mi(\tau)^2}, \tau$$

- **Area under precision recall curve (AUPR)**

$$\text{AUPR} = \int_0^1 \text{Precision}(R)\, dR$$

where Precision(R) represents the precision at a given recall level (R).

- **Recall@k**

$$\text{Recall@}k = \frac{1}{N} \sum_{i=1}^{N} \mathbf{1}\left[\exists j \in \mathcal{G}_i : j \in \text{topk}(s_i, k)\right]$$

where N is the number of queries (batch size), s_i is the similarity scores for the i-th query against all candidates, $\mathcal{G}_i$ is the set of ground truth indices for the i-th query, $\text{topk}(s_i, k)$ returns the indices of the k highest scoring candidates, and $\mathbf{1}[\cdot]$ is the indicator function that returns 1 if the condition is true, 0 otherwise.

- **Mean Reciprocal Rank**

$$\text{MRR} = \frac{1}{N} \sum_{i=1}^{N} \frac{1}{\min_{j \in \mathcal{G}_i} \text{rank}(j, s_i)}$$

where N is the number of queries, $\mathscr{G}_i$ is the set of ground truth indices for the i-th query, $\text{rank}(j, s_i)$ returns the position of candidate j in the sorted (descending) list of similarity scores s_i, and $\min_{j \in \mathscr{G}_i}$ finds the minimum rank among all ground truth indices.

Supplementary Note S3: Existing Methods

For full training, we compared our method to five baseline methods, namely Naive, DiamondBLAST [2, 5], TransFew [1], DeepGO-SE [4], and SPROF-GO [9]. Here's a concise overview of each method:

Naive: The Naive method simply uses the frequency of Gene Ontology (GO) terms in the training dataset to make predictions.

DiamondBLAST [2, 5] is based on sequence similarity scores obtained through BLAST, it identifies similar sequences from the training set and transfers annotations from the most similar ones .

SPROF-GO [9] is an alignment-free method employing a pre-trained protein language model to extract informative sequence embeddings. It utilizes self-attention pooling to focus on crucial residues and integrates homology information using a label diffusion algorithm . Test predictions were obtained through the provided web server for SPROF-GO.

DeepGO-SE [4] utilizes a pre-trained large protein language model combined with GO background knowledge and protein-protein interactions (PPIs) to make accurate predictions about protein functions. Predictions for DeepGO-SE were generated by cloning and running the tool locally.

TransFew [1] is a deep learning method for protein function prediction, with a focus on rare GO terms. It generates protein and GO term embeddings using pretrained models (ESM2 and BioBERT), and integrates them via cross-attention to transfer knowledge from common to rare terms and improve prediction accuracy.

Supplementary Note S4: Semantic Specificity of Zero-Shot GO Predictions

To further analyze the semantic characteristics of zero-shot predictions, we evaluated the distribution of information content (IC) of predicted Gene Ontology (GO) terms. Information content measures the specificity of a GO term, where higher IC values correspond to more specific functional annotations.

For each modality (Sequence, Structure, Text, and InterPro), we compared the IC distributions of correctly and incorrectly predicted GO terms across all three ontologies (BP, MF, and CC) and retrieval depths (R@1, R@3, and R@5). Figure S15 shows the resulting violin plots.

Across all ontologies, correctly predicted GO terms generally exhibit lower to moderate IC values compared to incorrect predictions. This indicates that FunBind more reliably predicts general or moderately specific functional annotations, while errors are more likely to occur for highly specific GO terms with higher IC values.

Increasing the retrieval depth from R@1 to R@5 increases the likelihood of capturing the correct annotation within the retrieved candidates, demonstrating that the model is capable of identifying relevant functional signals even when the most specific annotations are challenging to predict at the top rank.

Importantly, the distributions of correctly predicted terms extend beyond the lowest IC values, demonstrating that FunBind is not restricted to predicting only very generic GO terms. Instead, the model is capable of correctly predicting GO terms with moderate semantic specificity across different modalities and ontologies.

Overall, these results suggest that while zero-shot prediction of highly specific GO terms remains challenging, FunBind successfully captures meaningful functional signals that allow it to generalize beyond the training annotations and produce semantically informative predictions.

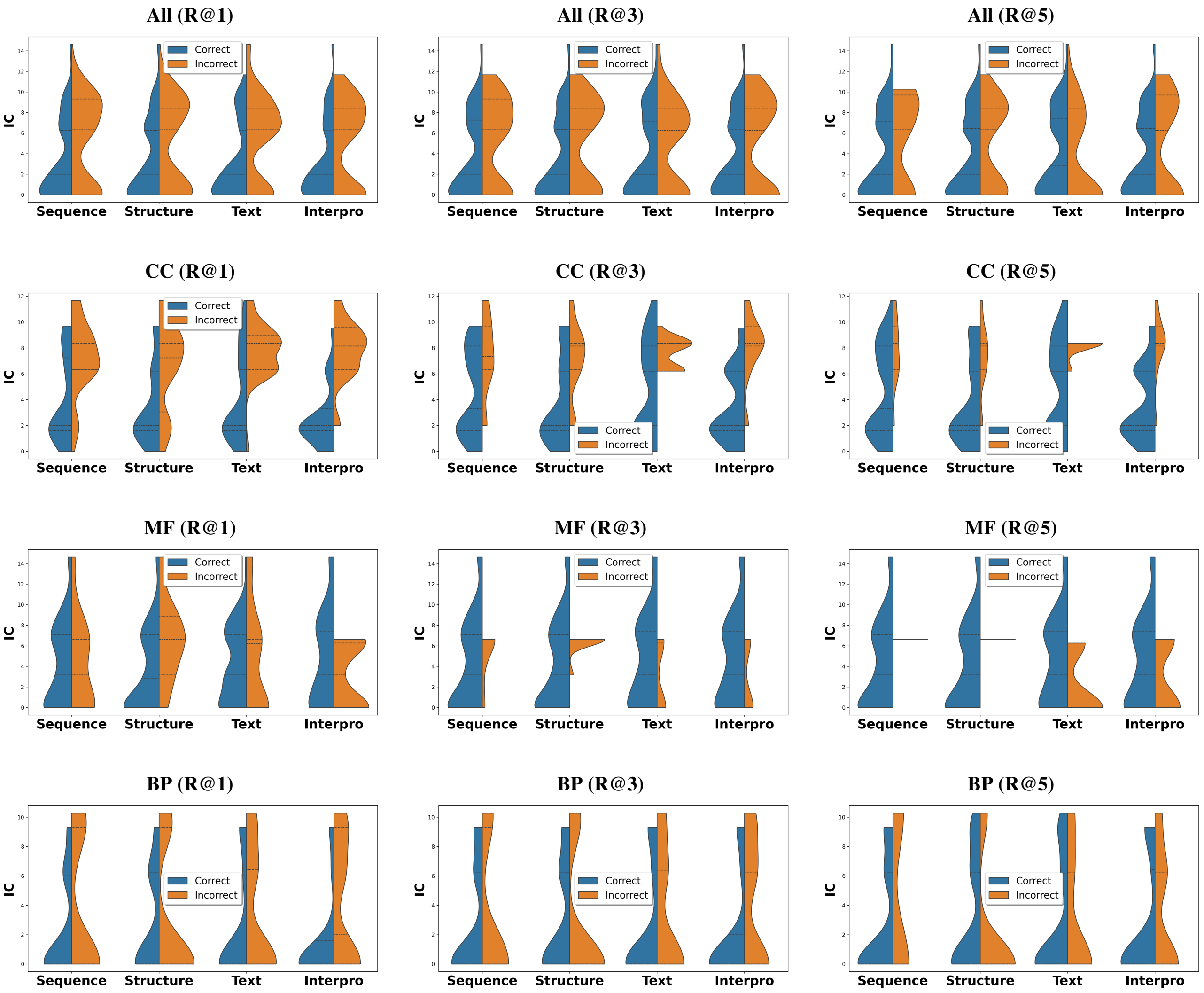

Figure S15: Distribution of information content (IC) for correctly (blue) and incorrectly (orange) predicted zero-shot GO terms across modalities and ontologies. Lower IC values correspond to more general GO terms, while higher IC values indicate more specific functional annotations. Across all ontologies, correct predictions tend to concentrate at lower to moderate IC values, whereas incorrect predictions are more frequent among highly specific GO terms. This trend improves with increasing retrieval depth (R@1 to R@5), indicating that more specific functional terms are increasingly captured among the top-ranked predictions.

Table S11: Performance of multimodal FunBind, pairwise modal models, and single-modal models on Test_All dataset. Bold font highlights the best performance and underline denotes the second best performance.

Methods		F_{max} (↑)			WF_{max} (↑)			$AUPR$ (↑)			S_{min} (↓)			Coverage		
		**CC**	**MF**	**BP**	**CC**	**MF**	**BP**	**CC**	**MF**	**BP**	**CC**	**MF**	**BP**	**CC**	**MF**	**BP**
**Single Modality**	Structure	0.671	0.648	0.413	0.526	0.563	0.335	0.659	0.546	0.299	5.352	3.801	15.660	0.91	0.90	0.90
	Interpro	0.657	0.643	0.421	0.505	0.546	0.344	0.668	0.552	0.313	5.862	3.977	15.670	0.95	0.94	0.93
	Sequence	0.695	0.663	0.449	0.557	0.570	0.375	0.516	0.491	0.330	5.413	3.853	15.472	1.0	1.0	1.0
	Text	0.692	0.677	0.441	0.546	0.586	0.366	0.713	0.585	0.334	5.339	3.691	15.145	0.98	0.98	0.98
**Pairwise Modality**	Structure Interpro	0.703	0.678	0.444	0.559	0.592	0.369	0.713	0.609	0.351	5.069	3.754	15.068	0.98	0.98	0.97
	Structure Sequence	0.711	0.681	0.458	0.579	0.592	0.387	0.692	0.594	0.361	5.067	3.618	15.080	1.0	1.0	1.0
	Structure Text	0.707	0.690	0.451	0.569	0.604	0.380	0.710	0.614	0.355	5.017	3.524	14.849	0.98	0.97	0.95
	Interpro Sequence	0.712	0.682	0.463	0.577	0.593	0.391	0.720	0.605	0.373	5.074	3.751	14.909	1.0	1.0	1.0
	Interpro Text	0.694	0.675	0.450	0.549	0.589	0.374	0.702	0.595	0.352	5.249	3.709	14.943	0.97	0.96	0.96
	Sequence Text	0.713	0.693	0.471	0.581	0.604	0.401	0.705	0.617	0.373	5.051	3.538	14.771	1.0	1.0	1.0
	FunBind	**0.724**	**0.699**	**0.475**	**0.596**	**0.611**	**0.404**	**0.745**	**0.637**	**0.389**	**4.824**	**3.474**	**14.626**	1.0	1.0	1.0

The results in Table S11 show that pairwise modality combinations consistently outperform individual modalities across the CC and MF ontologies, indicating that different biological modalities provide complementary signals for protein function prediction. In particular, combinations involving sequence features yield the largest improvements, suggesting that sequence representations serve as a strong backbone modality that synergizes effectively with other data sources.

Among the pairwise combinations, the sequence–text modality achieves the strongest performance, indicating that evolutionary sequence features and textual functional descriptions provide highly complementary information. Structural representations also contribute additional predictive signal, though the improvements are generally smaller compared to sequence-based combinations.

InterPro features capture protein domain information, which aligns well with sequence-derived representations and further improves prediction when integrated with sequence embeddings.

Despite these improvements, the full multimodal FunBind model consistently outperforms all pairwise combinations across evaluation metrics. This result demonstrates that integrating all modalities enables the model to capture complementary information that cannot be fully recovered by any pairwise subset.

Supplementary Note S5: Comparing Zero-Shot GO Predictions of FunBind and three baseline methods

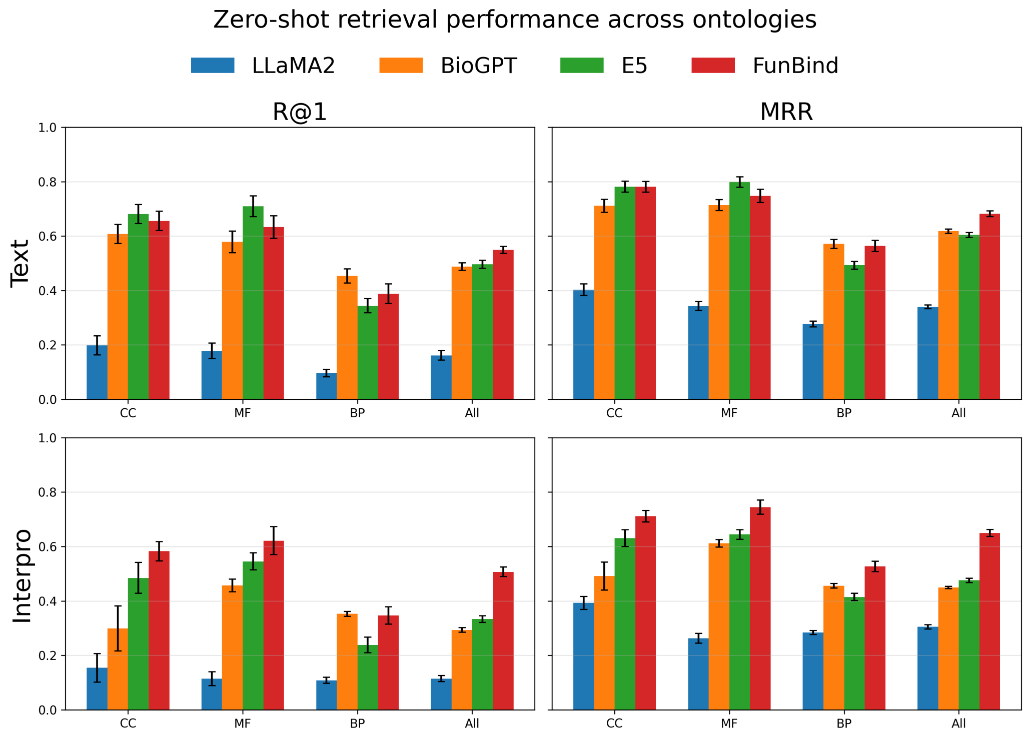

Figure S16: Performance (R@1, MRR) of FunBind and diffrent baseline for zero-shot protein function prediction across CC, MF, BP, and All. We compare general-purpose language models (LLaMA2), domain-specific (BioGPT), general embedding models (E5), and our aligned multimodal model (FunBind) using Text (top) and InterPro (bottom) modalities. FunBind consistently outperforms all baselines, highlighting the importance of cross-modal alignment

To further assess the performance of our zeroshot retrieval, we compare the performance of FunBind against three baselines: BioGPT [6], E5 [8], and LLaMA [7] (Figure S16). These text-based baselines are suitable since multiple modalities: Text, Interpro and Ontology in our framework are also text-based.

E5 is a retrieval-oriented embedding model pre-trained on a diverse mixture of multilingual text pairs using a contrastive objective similar to ours, resulting in well-structured embedding geometry. In contrast, BioGPT is pre-trained on biomedical corpora and thus captures domain-specific knowledge, providing strong domain alignment.

Performance varies substantially across three ontology branches and model families. Retrieval-optimized embeddings from E5 provide very strong zero-shot performance for Cellular Component and Molecular Function, where GO terms are relatively concise and semantically well-defined. In contrast, Biological Process is consistently more challenging, and biomedical language modeling with BioGPT becomes more competitive, suggesting that BP annotations rely more heavily on domain-specific textual semantics. FunBind achieves the strongest overall performance and is especially advantageous when InterPro modality is used.

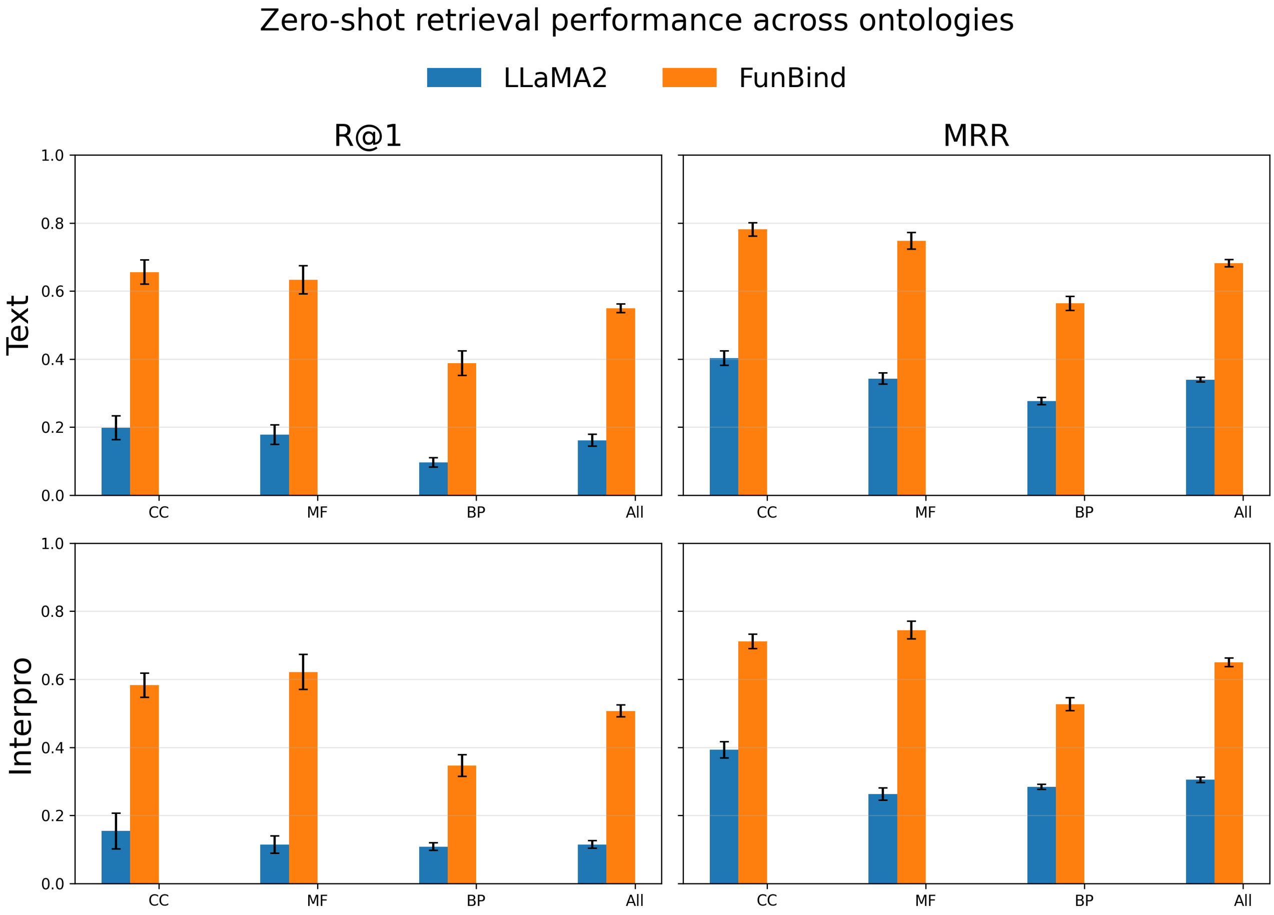

Figure S17: Ablation without contrastive learning. Zero-shot retrieval performance (R@1 and MRR) across GO ontologies using Text and InterPro modalities. Removing contrastive alignment significantly reduces performance, while FunBind consistently outperforms the non-aligned baseline, highlighting the importance of multimodal alignment.

Supplementary Note S6: Zero-shot Prediction on Non-homologous Proteins

To investigate this, we evaluate FunBind under a strict non-homologous setting by restricting the Test_Zero set to proteins sharing less than 30% sequence identity with any training protein.

The resulting non-homologous subset is relatively small due to the stringent filtering criteria; nevertheless, FunBind maintains strong retrieval performance across ontologies and modalities. The similarity matrices (Figure S18) reveal that FunBind produces well-structured alignment patterns, with high confidence concentrated along the correct protein–term correspondences.

Taken together, these results demonstrate that FunBind's zero-shot predictions are not solely driven by functional annotation transfer from closely related proteins. Instead, the model leverages learned representations that integrate sequence, structure, and textual information to generalize to unseen functions.

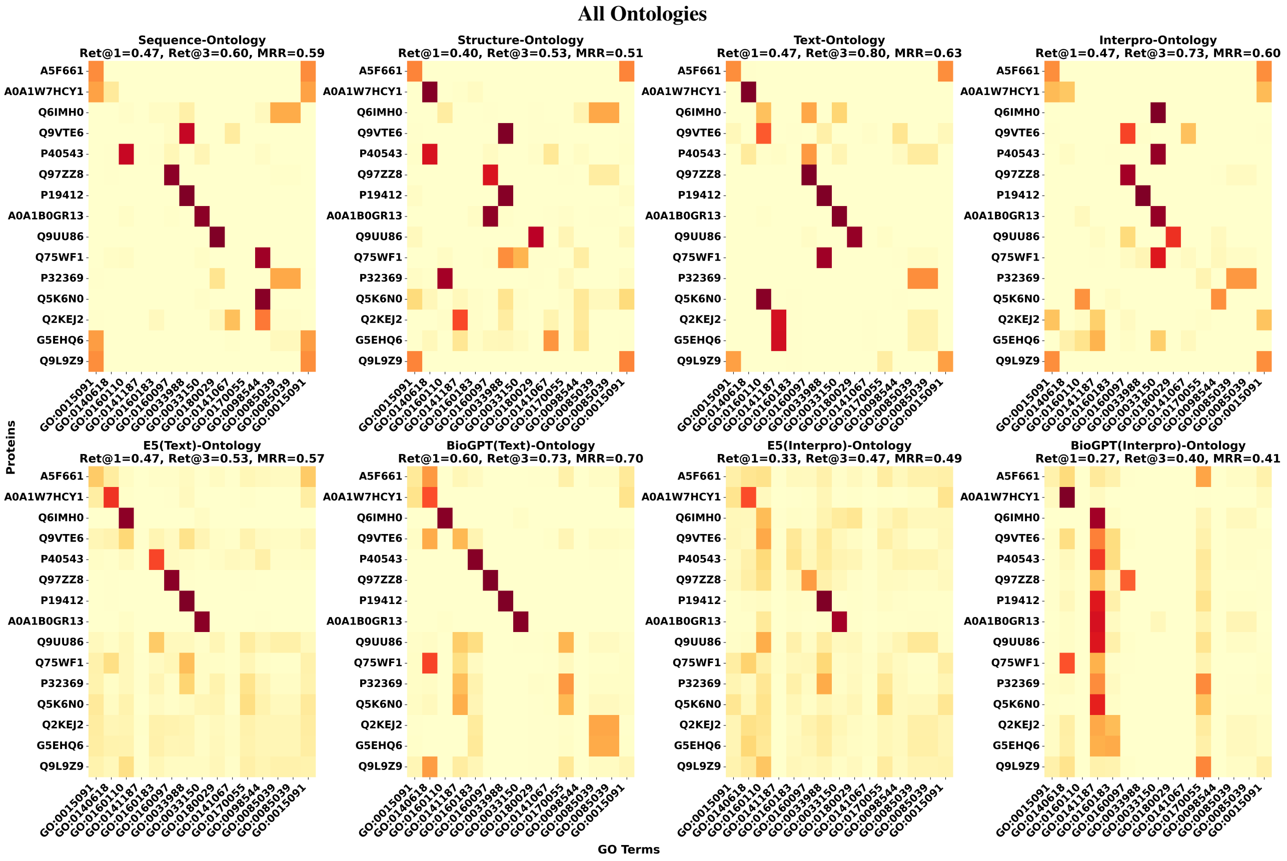

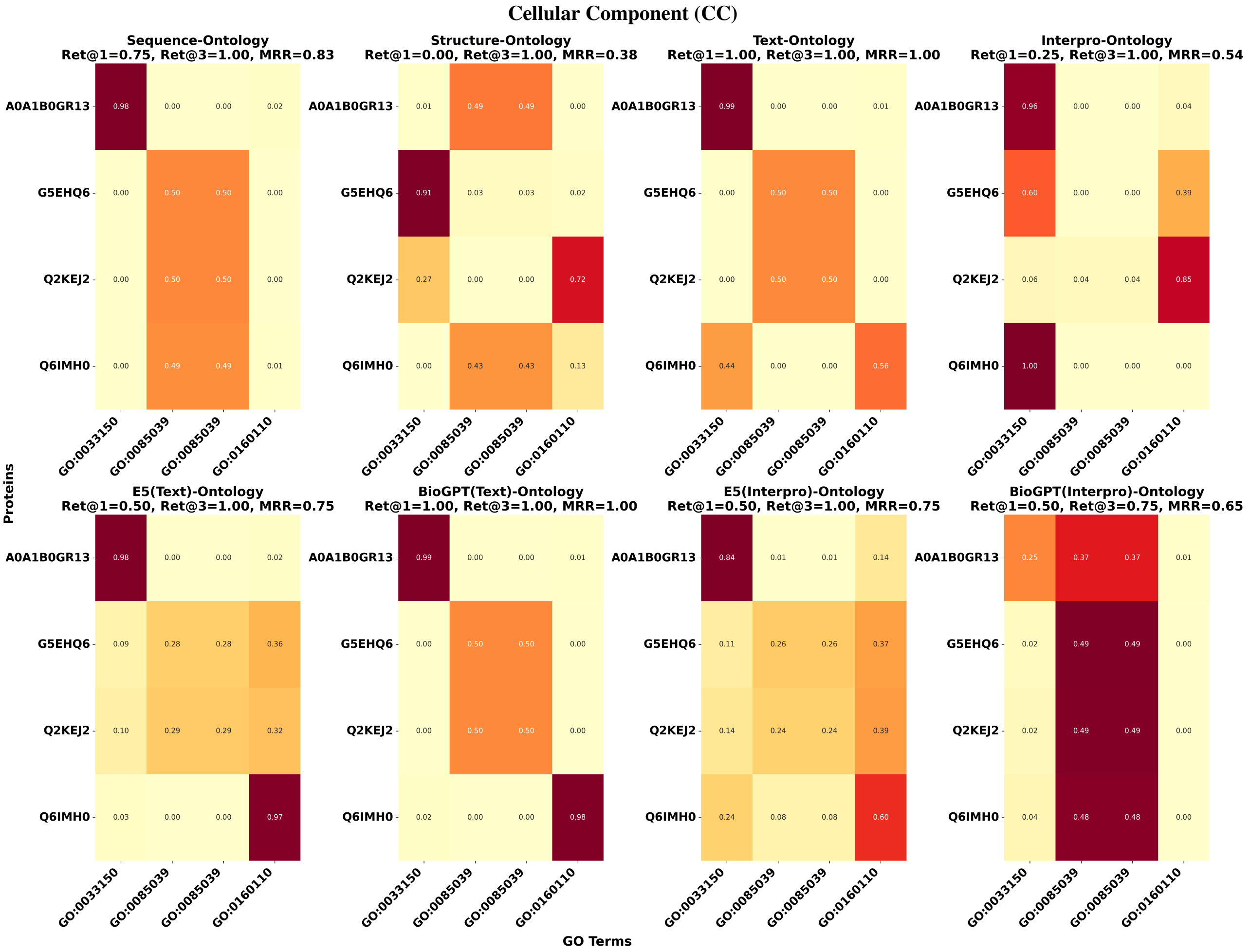
Cellular Component (CC)
Sequence-Ontology
Ret@1=0.75, Ret@3=1.00, MRR=0.83
Structure-Ontology
Ret@1=0.00, Ret@3=1.00, MRR=0.38
Text-Ontology
Ret@1=1.00, Ret@3=1.00, MRR=1.00
Interpro-Ontology
Ret@1=0.25, Ret@3=1.00, MRR=0.54
E5(Text)-Ontology
Ret@1=0.50, Ret@3=1.00, MRR=0.75
BioGPT(Text)-Ontology
Ret@1=1.00, Ret@3=1.00, MRR=1.00
E5(Interpro)-Ontology
Ret@1=0.50, Ret@3=1.00, MRR=0.75
BioGPT(Interpro)-Ontology
Ret@1=0.50, Ret@3=0.75, MRR=0.65
Proteins
GO Terms

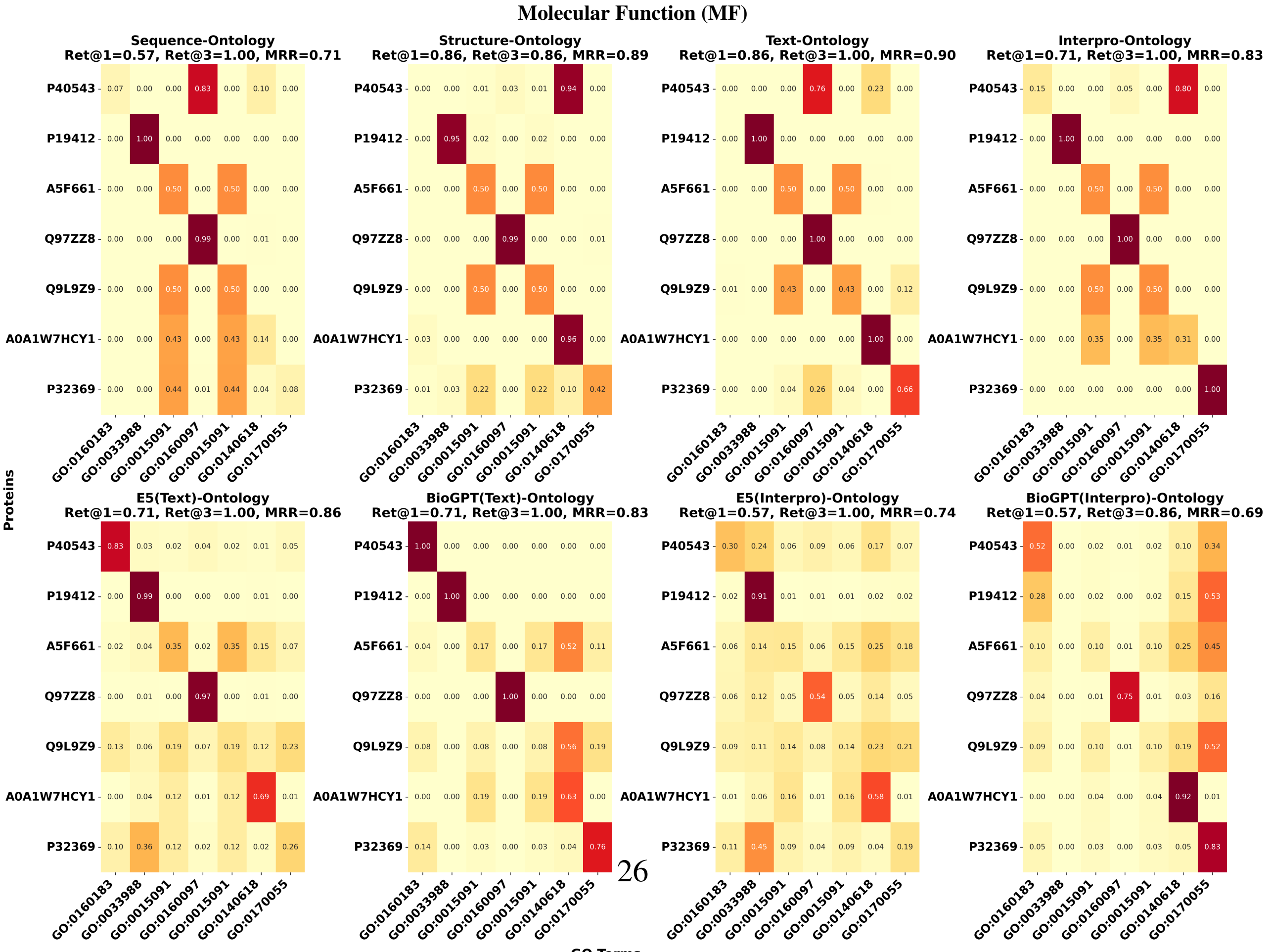
Molecular Function (MF)
Sequence-Ontology
Ret@1=0.57, Ret@3=1.00, MRR=0.71
Structure-Ontology
Ret@1=0.86, Ret@3=0.86, MRR=0.89
Text-Ontology
Ret@1=0.86, Ret@3=1.00, MRR=0.90
Interpro-Ontology
Ret@1=0.71, Ret@3=1.00, MRR=0.83
E5(Text)-Ontology
Ret@1=0.71, Ret@3=1.00, MRR=0.86
BioGPT(Text)-Ontology
Ret@1=0.71, Ret@3=1.00, MRR=0.83
E5(Interpro)-Ontology
Ret@1=0.57, Ret@3=1.00, MRR=0.74
BioGPT(Interpro)-Ontology
Ret@1=0.57, Ret@3=0.86, MRR=0.69
Proteins

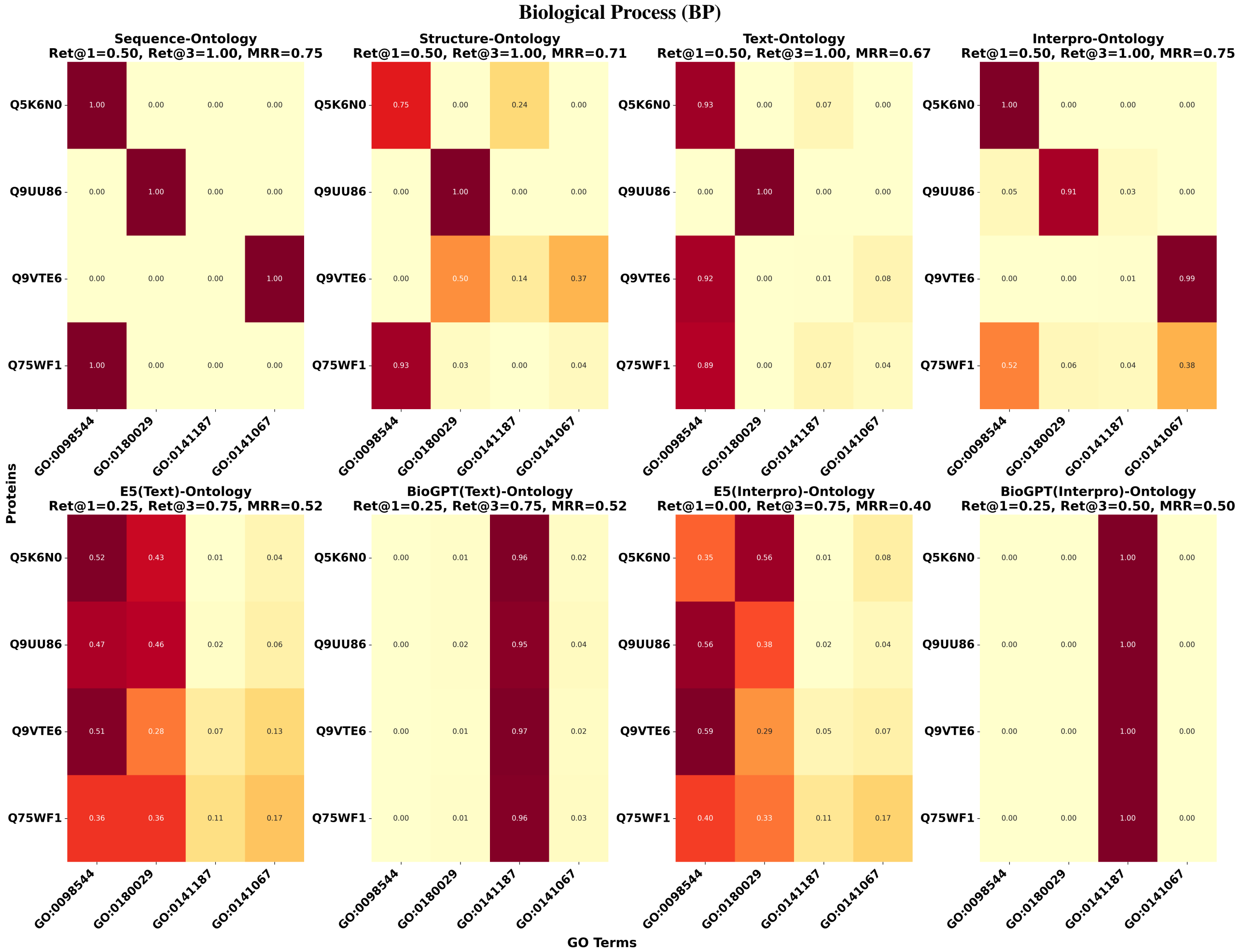

Figure S18: Zero-shot retrieval matrices on proteins with less than 30% sequence identity to the training set. FunBind (top row of each panel) produces structured alignment between proteins and GO terms, while baseline methods (E5 and BioGPT) show more diffuse or collapsed similarity patterns. These results demonstrate that FunBind maintains strong zero-shot performance even in the absence of homologous proteins.

References

Boadu, F., & Cheng, J. (2024, 08). Improving protein function prediction by learning and integrating representations of protein sequences and function labels. *Bioinformatics Advances*, *4*(1), vbae120. Retrieved from https://doi.org/10.1093/bioadv/vbae120 doi: 10.1093/bioadv/vbae120

Buchfink, B., Xie, C., & Huson, D. H. (2015). Fast and sensitive protein alignment using diamond. *Nature methods*, *12*(1), 59–60.

Jiang, Y., Oron, T. R., Clark, W. T., Bankapur, A. R., D'Andrea, D., Lepore, R., ... others (2016). An expanded evaluation of protein function prediction methods shows an improvement in accuracy. *Genome biology*, *17*(1), 1–19.

Kulmanov, M., Guzmán-Vega, F. J., Duek Roggli, P., Lane, L., Arold, S. T., & Hoehndorf, R. (2023). Deepgo-se: Protein function prediction as approximate semantic entailment. *bioRxiv*, 2023–09.

Kulmanov, M., & Hoehndorf, R. (2020). Deepgoplus: improved protein function prediction from sequence. *Bioinformatics*, *36*(2), 422–429.

Luo, R., Sun, L., Xia, Y., Qin, T., Zhang, S., Poon, H., & Liu, T.-Y. (2022). Biogpt: generative pre-trained transformer for biomedical text generation and mining. *Briefings in bioinformatics*, *23*(6), bbac409.

Touvron, H., Martin, L., Stone, K., Albert, P., Almahairi, A., Babaei, Y., ... others (2023). Llama 2: Open foundation and fine-tuned chat models. *arXiv preprint arXiv:2307.09288*.

Wang, L., Yang, N., Huang, X., Yang, L., Majumder, R., & Wei, F. (2024). Multilingual e5 text embeddings: A technical report. *arXiv preprint arXiv:2402.05672*.

Yuan, Q., Xie, J., Xie, J., Zhao, H., & Yang, Y. (2023). Fast and accurate protein function prediction from sequence through pretrained language model and homology-based label diffusion. *Briefings in bioinformatics*, *24*(3), bbad117.

Zhou, N., Jiang, Y., Bergquist, T. R., Lee, A. J., Kacsoh, B. Z., Crocker, A. W., ... others (2019). The cafa challenge reports improved protein function prediction and new functional annotations for hundreds of genes through experimental screens. *Genome biology*, *20*(1), 1–23.
